# Do Distinct Groups of Reactively and Proactively Aggressive Children Exist? A Confirmatory Latent Profile Approach

**DOI:** 10.1007/s10802-021-00813-0

**Published:** 2021-04-21

**Authors:** Anouk van Dijk, Julie A. Hubbard, Peter K. H. Deschamps, Wieteke Hiemstra, Hanneke Polman

**Affiliations:** 1 Department of Psychology, Utrecht University, Utrecht, The Netherlands; 2 Research Institute of Child Development and Education, University of Amsterdam, Amsterdam, The Netherlands; 3 Department of Psychological and Brain Sciences, University of Delaware, Newark, Delaware, USA; 4 Department of Psychiatry, University Medical Center Utrecht, Utrecht, The Netherlands

**Keywords:** Children, Aggressive behavior, Reactive motives, Proactive motives, Latent profile analysis

## Abstract

The present study examined whether there are distinct groups of children with reactive versus proactive motives for their aggressive behavior. We extended previous research by using a person-based analytical approach on data from a questionnaire assessing children’s motives independently from the severity of their aggression. Two competing hypotheses were tested. The *both subtypes hypothesis* holds that both reactive and proactive subtypes exist, as well as a mixed subtype. The *reactive only hypothesis* holds that only reactive and mixed subtypes exist. Hypotheses were tested on existing data from a community sample of children displaying aggression (Study 1: *n* = 228, ages 10–13, 54% boys), and two clinical samples of children with aggressive behavior problems (Study 2: *n* = 115, ages 8–13, 100% boys; Study 3: *n* = 123, ages 6–8, 78% boys). Teachers reported on children’s reactive and proactive motives. We selected measures available from peers, parents, teachers, and children themselves to compare the supported subtypes on variables that previous literature suggests uniquely correlate with reactive versus proactive aggression. Confirmatory latent profile analyses revealed that the *both subtypes hypothesis* best fit the data of all three samples. Most children were classified as reactive (55.7–61.8% across samples), with smaller percentages classified as proactive (10.4–24.1%) and mixed (18.0–33.9%). However, these subtypes only differed in expected directions on 7 out of 34 measures. Overall, results support the existence of both reactive and proactive subtypes of aggressive children, but the distinctiveness of these subtypes in terms of social-emotional characteristics warrants further study.

**Supplementary Information:**

The online version contains supplementary material available at 10.1007/s10802-021-00813-0.

Children behave aggressively for different reasons. They may aggress when feeling threatened or provoked, or they may aggress to obtain a desired outcome (Dodge et al., 1997). This distinction between reactive and proactive motives has proven useful when describing different reasons why children aggress (e.g., Card & Little, 2006; Polman et al., 2007). Researchers debate, however, whether this distinction is also useful when describing groups of aggressive children (Smeets et al., 2017). Do distinct groups of aggressive children exist, with one group aggressing for reactive reasons and the other for proactive reasons? Addressing this question will advance our knowledge of aggression in middle childhood and may help practitioners tailor preventive interventions to children’s individual needs (Vitaro et al., 2006). This is the goal of the present study, and we address it using existing data from a community sample and two clinical samples.

From a developmental perspective, if distinct groups of reactively and proactively aggressive children were to exist, these groups are likely to emerge in middle childhood. Although longitudinal research tracking reactive and proactive aggression from early to middle childhood is lacking, both theory and empirical findings suggest that the subtypes of aggression have distinct developmental underpinnings. Theoretically, reactive aggression is considered an impulsive, emotional, defensive response to perceived threat (Dodge et al., 1997) explained by the frustration-aggression hypothesis (Berkowitz, 1989) and temperamental theories emphasizing negative emotionality and lack of self-control (Moore et al., 2018). Proactive aggression, in contrast, is described as unemotional, reward-driven behavior (Dodge et al., 1997) that has been explained by social-learning models of aggression (Bandura, 1978) and temperamental theories emphasizing low emotional reactivity to aversive stimuli (Frick et al., 2003). Although speculative, these theories suggest that reactive aggression may emerge in early childhood, whereas proactive aggression may not emerge until middle childhood, given that the development of proactive aggression requires children to observe aggressive role models and learn that aggression may help them meet their goals (Vitaro et al., 2006). For this reason, we focus our research on middle childhood.

Empirically, support for distinct groups of reactive versus proactive aggressive children is found in the literature on the differential correlates of reactive and proactive aggression (e.g., Card & Little, 2006; Dodge & Coie, 1987; Frick et al., 2003; Hubbard et al., 2010; Kimonis et al., 2006; Raine et al., 2006; Vitaro et al., 2006). Children with high levels of reactive aggression are more likely to experience peer rejection or victimization, attribute hostile intent to others, and display anger regulation difficulties, social anxiety, internalizing problems, and ADHD symptoms. Children with high levels of proactive aggression, in contrast, are more likely to have instrumental goals, engage in delinquency, and display conduct problems, empathy deficits and psychopathic traits. These findings suggest that specific cognitive, emotional, social, or psychological characteristics may predispose children to either reactive or proactive motives for their aggression.

Some researchers, however, have argued that unique correlates of reactive and proactive aggressive *behavior* do not necessarily support the existence of distinct subtypes of reactive and proactive aggressive *children* (Smeets et al., 2017). Correlates of reactive and proactive aggression may co-exist within individual children (e.g., they may display anger *and* conduct problems). Indeed, meta-analyses have found strong correlations between reactive and proactive aggression (Card & Little, 2006; Polman et al., 2007). Furthermore, studies which have approached this question using median splits or cut-off scores fail to provide a clear answer, as by definition, these approaches result in four groups of children (low on both reactive and proactive aggression, high on both, or low on one and high on the other; e.g., Carroll et al., 2018; Dodge & Coie, 1987; Salmivalli & Nieminen, 2002).

More recently, researchers have directly tested the existence of distinct reactive and proactive subtypes of aggressive youth using person-based analyses (e.g., cluster analysis or latent profile analysis). These studies have typically identified reactive groups with high reactive motives and low proactive motives, as well as mixed groups with similar levels of reactive and proactive motives, but no proactive group with high proactive motives and low reactive motives (Colins, 2016; Cui et al., 2016; Euler et al., 2017; Marsee et al., 2014; Muñoz et al., 2008; Pang et al., 2013; Smeets et al., 2017; Thomson & Centifanti, 2018). One study did identify a “proactive” group with higher proactive motives compared to other subgroups (Mayberry & Espelage, 2007), but these children still had higher reactive than proactive motives. Thus, overall, findings from person-based analytical studies support the existence of reactive and mixed, but not proactive, subtypes of aggressive children.

However, an important limitation of the person-based analytical studies conducted thus far is that they all used questionnaires with a limited ability to discriminate between reactive and proactive motives. Items on these questionnaires tend to describe the same form of aggression (e.g., hitting) to assess either reactive motives (e.g., hits when angry) or proactive motives (e.g., hits for fun), resulting in high intercorrelations between reactive and proactive subscales (Card & Little, 2006; Polman et al., 2007). Newer questionnaires have been developed to address this confound, and they successfully disentangle reactive from proactive motives, as evidenced by low, nonsignificant intercorrelations between reactive and proactive subscales (Little et al., 2003; Polman et al., 2009). Yet, these questionnaires have never been combined with a person-based analytical approach.

As such, the question of whether distinct groups of reactive and proactive aggressive children exist remains unanswered and is the focus of the current study. We extended previous research by using (a) a person-based analytical approach and (b) data from a questionnaire designed to assess motives independently from the frequency of aggression. Hypotheses were tested on existing data from three middle childhood samples: Study 1 uses a community sample of children displaying aggression (ages 10–13; 54% boys); Study 2 extends findings to a clinical sample with aggressive behavior problems (ages 8–13; 100% boys); and Study 3 extends findings to a younger clinical sample (ages 6–8; 78% boys) to test if distinct subgroups have emerged at the start of middle childhood. These data provide a unique opportunity to draw more finite conclusions regarding the existence of distinct reactive and proactive subtypes of aggressive children.

Given the wealth of research reviewed above, we adopted a confirmatory hypothesis testing approach. Two competing hypotheses arise from the literature. Research focusing on distinct correlates suggests that both reactive and proactive subtypes exist, as well as a mixed subtype. We termed this the *both subtypes hypothesis*. Research using a person-based analytical approach has found no evidence for the proactive subtype, suggesting that only reactive and mixed subtypes exist. We labeled this the *reactive only hypothesis*. We further differentiated between *pure* and *predominant* versions of each hypothesis. The *pure* version states that, if children are classified into the reactive or proactive subtype, their score on the other motive must be 0 (Table 1). The *predominant* version holds that, if children are classified into the reactive or proactive subtype, their score on the other motive must be substantially lower, although it may be greater than 0. We expressed this balance as a ratio, classifying children into the reactive subtype if their reactive motives were 1.5 times stronger than their proactive motives, and classifying children into the proactive subtype if their proactive motives were 1.5 times stronger than their reactive motives.^1^ By defining *pure* and *predominant* versions of each hypothesis, we aimed to provide a fair test of both hypotheses.

**Table 1:** Subgroup Constraints on Reactive (Re) and Proactive (Pro) Motive Scores for the Pure and Predominant Versions of the Reactive Only and Both Subtypes Hypotheses

	Reactive subgroup	Mixed subgroup	Proactive subgroup
Reactive only – pure	Re > 0; Pro = 0	Re > 0; Pro > 0	
Reactive only – predominant	Re > Pro*1.5	Re ≤ Pro*1.5	
Both subtypes – pure	Re > 0; Pro = 0	Re > 0; Pro > 0	Re = 0; Pro > 0
Both subtypes – predominant	Re > Pro*1.5	Highest / lowest ≤ 1.5^a^	Pro > Re*1.5

^a^The mixed subgroup of the *both subtypes – predominant* hypothesis includes children for whom the ratio between their highest and lowest motive score is less than or equal to 1.5

The main goal of this study was to test our competing hypotheses on three datasets that all assessed children’s motives independently from the severity of their aggression (i.e., using the Instrument for Reactive and Proactive Aggression [IRPA]; Polman et al., 2009). A secondary goal was to compare the subgroups of the supported hypothesis on a set of variables that discriminate between reactive and proactive motives. We used the theoretical frameworks and empirical findings discussed above to select measures included in the datasets that should uniquely correlate with reactive and proactive aggression. Given reactive aggression’s links to emotionality, impulsivity, and social difficulties, we predicted that the reactive and mixed subgroups would score higher than the (possible) proactive subgroup on ADHD symptoms, emotional symptoms, peer problems, trait anxiety, provoked anger, hostile intent attribution, anger attribution bias, and victimization, but lower on social acceptance, social preference, and popularity. In contrast, given proactive aggression’s links to unemotionality and goal orientation, we predicted that the proactive and mixed subgroups would score higher than the reactive subgroup on conduct problems, psychopathy, dominance, aggression approval, bullying, and coercive strategy use, but lower on empathy and empathic responding. As the theoretical underpinnings of reactive and proactive aggression are the same across ages and aggression severity, we tested these predictions for available measures in all three samples. Last, as an exploratory goal, we examined gender differences in our reactive, mixed, and (possibly) proactive subgroups, and found none (see Appendix S5 in the Supplementary Information for a detailed description of these analyses).

## Study 1: Community Sample

### Method

#### Participants

 Data were used from a validation study of the IRPA (Polman et al., 2009). The sample included *N* = 427 children recruited in 2007 from 22 fifth- and sixth-grade Dutch classrooms. Schools distributed passive consent letters to parents or caretakers—a procedure in line with Dutch university regulations at the time of data collection. Parents could withdraw their consent by returning the letter to schools (around 3% did). This study was not reviewed by an ethics board. Following Polman and colleagues (2009), we excluded children with incomplete scores on reactive or proactive motives (*n* = 197). Such missing scores are created by design of the questionnaire, because children whose teachers reported that they did not display aggressive behavior could not receive ratings on their motives. We also excluded data for one classroom in which only two children had complete motive scores, so we could conduct multilevel analyses. The final sample consisted of *n* = 228 children ages 10–13 (54% boys; *M*_age_ = 11.68, *SD* = 0.70).

#### Instrument for Reactive and Proactive Aggression (IRPA)

 Teachers rated the frequency of seven forms of aggression (i.e., kicking, pushing, hitting, name calling, arguing, gossiping, doing sneaky things) within the past month on a 5-point Likert scale (0 = *never*, 1 = *once or twice*, 2 = *weekly*, 3 = *several times a week*, 4 = *daily*). Frequency of aggression was calculated as the average across these items. For each aggression item that occurred at least once (score > 0), teachers rated three items on reactive motives (e.g., “because this child was angry”) and three items on proactive motives (e.g., “because this child takes pleasure in it”) on a 5-point Likert scale (0 = *never*, 1 = *rarely*, 2 = *sometimes*, 3 = *most of the time*, 4 = *always*). If aggression items never occurred (score = 0), motive items were coded as missing. First, we averaged motives across forms, resulting in three reactive and three proactive scores per child. Second, we averaged these scores to create the reactive and proactive motive scales. High scores on motive scales thus reflect that children often had this motive for their aggressive behavior, regardless of how frequently they displayed aggression.

Previous research suggests that teachers are reliable informants regarding the motives for children’s aggression (Dodge & Coie, 1987) and has reported good discriminant, convergent and construct validity for the IRPA (Polman et al., 2009). Reactive and proactive motive scales were not correlated with each other (*r* = 0.03), moderately correlated with a conventional reactive-proactive aggression measure (Dodge & Coie, 1987), and uniquely correlated with most expected variables (i.e., reactive motives with trait anxiety, social acceptance, emotional problems, peer problems, victimization, and popularity, and proactive motives with conduct problems, coercive strategy use, bullying, and bossiness). In the current sample, internal consistency reliability of the IRPA was good for all subscales (aggression: α = 0.76; reactive motives: *r* = 0.45; proactive motives: *r* = 0.46),^2^ and the reactive and proactive motive scales were not correlated (*r* = -0.003, *p* = 0.960).

### Subgroup Comparison Measures

#### Teacher-report Measures: ADHD Symptoms, Emotional Symptoms, Conduct Symptoms, and Peer Problems

 Teachers completed the Strengths and Difficulties Questionnaire (Goodman, 2001). We included the four 5-item problem scales, rated on a 3-point scale from 1 (*not true*) to 3 (*certainly true*). Following Polman and colleagues (2009), we removed the item “often loses temper” from the conduct problems scale. Scores were averaged across items. Reliability was sufficient for all scales: *ADHD symptoms* (α = 0.84), *emotional symptoms* (α = 0.81), *conduct problems* (α = 0.65), *peer problems* (α = 0.79).

#### Self-report Measures: Trait Anxiety, Psychopathy, Dominance, Empathy, and Social Acceptance

 Children completed 5 self-report questionnaires. *Trait anxiety* was assessed using the 20-item Trait Anxiety subscale from the State-Trait Anxiety Inventory for Children (Spielberger et al., 1970). Items were rated on a 3-point scale from 0 (*almost never*) to 2 (*often*). *Psychopathy* was assessed using a 20-item version of the Youth Psychopathic Traits Inventory – Child Version (Van Baardewijk et al., 2008). Items were rated on a 4-point scale from 0 (*does not apply at all*) to 3 (*applies very well*). *Dominance* was assessed using the Dominance subscale from the Dutch Personality Questionnaire-Junior (Luteijn et al., 1989). This subscale includes 15 statements about the self which are rated on a 3-point scale (0 = *no*; 1 = *?*; 2 = *yes*). *Empathy* was assessed using the Empathic Sadness subscale from the Empathy Index for Children and Adolescents (Bryant, 1982). This subscale includes 7 items which are rated as 0 (*no*) or 1 (*yes*). *Social acceptance* was assessed using the 6-item Social Acceptance subscale of Harter’s Perceived Competence Scale (Harter, 1982). Items were rated on a 4-point scale from 0 (*not at all true*) to 3 (*absolutely true*). Scores for all measures were calculated as the average across items, with higher scores indicating higher levels of the trait. Reliability was sufficient to good for all measures: *trait anxiety* (α = 0.85), *psychopathy* (α = 0.87), *dominance* (α = 0.67), *empathy* (α = 0.80), and *social acceptance* (α = 0.81).

#### Social Information Processing Measures: Provoked Anger, Hostile Intent Attribution, and Aggression Approval

 Children answered several questions following 4 gender-matched vignettes describing ambiguous peer provocations (De Castro et al., 2005). *Provoked anger* was assessed using a 7-point scale ranging from 0 (*not angry at all*) to 6 (*very angry*). *Hostile intent attribution* was assessed using one question (i.e., “Was it an accident or did he/she do it on purpose?”) rated on a 7-point scale ranging from 0 (*totally by accident*) to 6 (*totally on purpose*). *Aggression approval* was assessed using three aggressive response options for each vignette rated on a 7-point scale ranging from 0 (*not good at all*) to 6 (*very good*). We averaged across vignettes to create scales for *provoked anger* (α = 0.78), *hostile intent attribution* (α = 0.69) and *aggression approval* (α = 0.94).

#### Peer Nomination Measures: Social Preference, Coercive Strategy Use, Bullies Others, Victimized, Angry Easily, and Popular

 Children received a class roster and nominated an unlimited number of classmates fitting each description. Nominations were *z*-standardized within classroom. *Social preference* was assessed using children’s nominations of classmates they liked most and least. Scores were calculated following standard procedures (Coie et al., 1982). *Coercive strategy use* was assessed using 6 items (Hawley, 2003). Scores were averaged across items (α = 0.89). For the other measures, children were asked to nominate classmates who fit each of the following descriptions: (1) *bullies others*, (2) *victim* of bullying, (3) gets *angry easily*, and (4) is *popular*. These were single-item scores.

#### Analytical Approach

 Our main goal was to examine whether there are distinct subtypes of children with reactive versus proactive motives for their aggressive behavior. We described four hypotheses covering the main perspectives in the extensive literature and used confirmatory latent profile analyses (LPA) in Mplus (version 8) to select which hypothesis best fit the data (Finch & Bronk, 2011). A major advantage of confirmatory LPA compared to standard LPA is that we could compare reactive and proactive motives *within* rather than *between* children. That is, children were classified as one subtype if their score on that motive exceeded their own score on the other motive. This provides a more precise answer to our research question than standard LPA, which classifies children as the subtype for which their scores exceeded the sample mean. We specified a model for each hypothesis based on predefined constraints (Table 1) and assessed each model’s fit using a random intercept on the latent class indicator to account for clustering within classrooms. We compared the fit of these non-nested models using AIC, BIC, and sample size adjusted BIC (aBIC) and assessed classification quality using entropy. We selected the model with the lowest AIC, BIC, and aBIC, and sufficient entropy (i.e., entropy > 0.80) as the best-fitting model. As our choice for defining ‘predominant’ may affect the results, we conducted sensitivity analyses for different constraints (i.e., besides a ratio of 1.50, we used the ratios of 1.25, 1.75 and 2.00). These analyses yielded the same overall conclusions (Appendix S1, Supplementary Information).

As a secondary goal, we compared the subgroups of the best-fitting hypothesis on variables that literature suggests uniquely correlate with reactive and proactive aggression. For these analyses, we classified children into reactive, mixed, and possibly proactive groups by applying the constraints of the supported hypothesis to their reactive and proactive scores (e.g., for the *reactive-only pure hypothesis*, a child with a reactive score of 0.5 and a proactive score of 0 would be classified in the reactive group). This approach differs from previous studies using exploratory LPA (e.g., Smeets et al., 2017). Because those studies had no pre-defined constraints, they created subgroups using output from the LPA specifying the group to which each child had the highest probability of belonging. With confirmatory LPA, this approach is not necessary because the class constraints are defined a priori. Thus, we used LPA to determine which model best fit the data, and then applied the constraints of the supported model to create the subgroups. We tested for subgroup differences using multivariate analysis of variance (MANOVA). Because most variables were skewed, we used bias-corrected accelerated (BCa) bootstrap 95% confidence intervals (5,000 samples).

### Results

First, we tested which hypothesis best fit the data. LPA results suggested that the *both subtypes – predominant model* outperformed all other models, as indicated by the lowest AIC, BIC and aBIC and highest entropy (Table 2). The multi-level results revealed no effects of classroom, as indicated by the non-significant variances of the random intercepts of the latent class indicator. Thus, the three-group model including a predominantly reactive, predominantly proactive, and mixed subgroup best fit the data for our community sample.

**Table 2:** Fit Indices for the Hypothesized Models for Studies 1–3

	AIC	BIC	aBIC	Entropy
Study 1				
Reactive only – pure	982.15	1006.15	983.97	0.811
Reactive only – predominant	947.81	975.25	949.89	0.851
Both subtypes – pure	941.75	976.04	944.35	0.823
Both subtypes – predominant	904.69	945.84	907.81	0.875
Study 2				
Reactive only – pure	608.29	624.76	605.79	0.679
Reactive only – predominant	585.93	605.14	583.02	0.744
Both subtypes – pure	601.56	623.52	598.24	0.844
Both subtypes – predominant	585.92	613.37	581.76	0.793
Study 3				
Reactive only – pure	597.05	613.92	594.95	n.a.^×^
Reactive only – predominant	583.01	602.69	580.56	0.751
Both subtypes – pure	600.56	623.06	597.76	n.a.^×^
Both subtypes – predominant	579.17	607.29	575.68	0.786

^×^No children were classified as purely reactive

We applied the constraints of this best-fitting hypothesis to create the subgroups. We found that 57.9% of children were classified as predominantly reactive, with higher scores on reactive motives (*M* = 1.44, *SD* = 0.76) versus proactive motives (*M* = 0.21, *SD* = 0.21). Another 24.1% of children were classified as predominantly proactive, with higher scores on proactive motives (*M* = 1.10, *SD* = 0.55) versus reactive motives (*M* = 0.20, *SD* = 0.30). The remaining 18.0% were classified as mixed, with similar scores on reactive (*M* = 1.26, *SD* = 0.58) and proactive motives (*M* = 1.24, *SD* = 0.64). Thus, even though reactive motives were on average more prevalent (*M* = 1.11, *SD* = 0.83) than proactive motives (*M* = 0.61, *SD* = 0.66), we still obtained a subgroup with predominantly proactive motives for their aggression. Last, we found that the subgroups differed in their frequency of aggression, *F*(2, 225) = 5.95, *p* = 0.003, η_p_^2^ = 0.05. The bootstrap 95% confidence intervals revealed that scores were higher in the mixed subgroup (*M* = 0.59, *SD* = 0.53, CI[0.44; 0.75]) compared to the reactive (*M* = 0.37, *SD* = 0.27, CI[0.33; 0.42]) and proactive subgroups (*M* = 0.49, *SD* = 0.43, CI[0.39; 0.60]), which did not significantly differ from each other.

The first column of Table 3 includes all subgroup comparison measures, and the second column states the hypothesized effect for each measure. The remaining columns display the descriptive statistics for the predominantly reactive, predominantly proactive, and mixed subgroups. A MANOVA revealed an overall effect of subgroup across these measures, *F*(36, 400) = 2.79, *p* < 0.001, η_p_^2^ = 0.20. The bootstrap 95% confidence intervals (Table 3) indicate that hypotheses were at least partially supported for the constructs of emotional symptoms, conduct problems, victimization, coercive strategy use, and bullying. However, no support emerged for the remaining constructs. Thus, these data provide partial support for the distinctiveness of the subgroups emerging from the *both subtypes – predominant model*.

**Table 3:** Hypothesized Mean Differences (*H*_*A*_), Range, Means (*M*), Standard Deviations (*SD*), and Bootstrap 95% Confidence Intervals (95% CI) of the Subgroup Comparison Measures for Children in the Predominantly Reactive (R), Proactive (P) and Mixed (M) Subgroups for Studies 1–3

Study 1												
			R (*n* = 132)		M (*n* = 55)		P (*n* = 41)		Reactive	Mixed	Proactive	η_p_^2^
	*H*_*A*_	Range	*M*	*SD*	*M*	*SD*	*M*	*SD*	95% CI	95% CI	95% CI	
Teacher-report												
ADHD symptoms	R,M > P	0.00–2.00	0.62^a^	0.52	0.91^b^	0.64	0.60^ab^	0.54	[0.52; 0.70]	[0.72; 1.11]	[0.46; 0.75]	0.04^*^
Emotional symptoms	R,M > P	0.00–1.60	0.42^a^	0.48	0.29^ab^	0.31	0.15^b^	0.29	[0.34; 0.50]	[0.20; 0.38]	[0.08; 0.23]	0.07^*^
Conduct problems	P,M > R	0.00–2.00	0.19^a^	0.26	0.49^b^	0.48	0.32^ab^	0.37	[0.14; 0.23]	[0.35; 0.64]	[0.23; 0.42]	0.11^*^
Peer problems	R,M > P	0.00–2.00	0.41	0.47	0.45	0.45	0.30	0.31	[0.34; 0.49]	[0.32; 0.58]	[0.23; 0.38]	0.02
Self-report												
Trait anxiety	R,M > P	0.00–1.90	0.54	0.36	0.52	0.28	0.47	0.25	[0.48; 0.61]	[0.44; 0.61]	[0.41; 0.54]	0.01
Psychopathy	P,M > R	0.00–2.35	0.61	0.39	0.57	0.46	0.63	0.50	[0.54; 0.68]	[0.44; 0.71]	[0.51; 0.76]	< 0.01
Dominance	P,M > R	0.13–1.73	0.75	0.30	0.76	0.27	0.80	0.37	[0.70; 0.80]	[0.68; 0.84]	[0.71; 0.89]	< 0.01
Empathy	P,M < R	0.00–1.00	0.33	0.29	0.31	0.26	0.42	0.32	[0.28; 0.38]	[0.24; 0.39]	[0.33; 0.50]	0.02
Social acceptance^×^	R,M < P	0.00–3.00	0.89	0.65	0.98	0.60	0.93	0.67	[0.79; 1.00]	[0.85; 1.12]	[0.65; 1.30]	0.02
Social information processing												
Provoked anger^×^	R,M > P	0.00–6.00	4.03^a^	1.50	3.27^b^	1.51	3.72^ab^	1.55	[3.75; 4.28]	[2.79; 3.74]	[3.31; 4.15]	0.04^*^
Hostile intent attribution^×^	R,M > P	0.00–6.00	2.85^a^	1.42	2.12^b^	1.15	2.55^ab^	1.51	[2.60; 3.10]	[1.78; 2.48]	[2.17; 2.93]	0.04^*^
Aggression approval	P,M > R	0.00–5.58	1.15	1.27	0.81	1.03	1.11	1.36	[0.94; 1.37]	[0.53; 1.14]	[0.78; 1.48]	0.01
Peer nomination												
Victimized	R,M > P	-1.55–4.56	0.32^a^	1.30	-0.17^b^	0.60	-0.15^b^	0.67	[0.11; 0.54]	[-0.34; 0.01]	[-0.31; 0.03]	0.05^*^
Angry easily	R,M > P	-1.00–4.18	0.22	1.20	0.47	1.30	0.25	0.99	[0.03; 0.42]	[0.08; 0.99]	[0.00; 1.52]	0.01
Social preference	R,M < P	-3.10–2.35	-0.08	1.07	-0.45	0.99	-0.32	0.96	[-0.26; 0.10]	[-0.76; -0.16]	[-0.57; -0.08]	0.02
Popular	R,M < P	-1.18–3.18	-0.06	0.97	0.13	0.86	0.21	1.08	[-0.22; 0.11]	[-0.12; 0.39]	[-0.07; 0.49]	0.01
Coercive strategy use	P,M > R	-0.79–3.43	0.05^a^	0.81	0.49^b^	0.98	0.39^ab^	0.97	[-0.07; 0.19]	[0.21; 0.81]	[0.15; 0.64]	0.04^*^
Bullies others	P,M > R	-0.97–3.47	-0.02^a^	0.90	0.54^b^	1.15	0.59^b^	1.24	[-0.16; 0.14]	[0.21; 0.91]	[0.28; 0.91]	0.07^*^

Study 2												
			R (*n* = 64)		M (*n* = 39)		P (*n* = 12)		Reactive	Mixed	Proactive	η_p_^2^
	*H*_*A*_	Range	*M*	*SD*	*M*	*SD*	*M*	*SD*	95% CI	95% CI	95% CI	
Psychopathy^×^	P,M > R	0.25–1.65	0.80	0.31	1.00	0.37	0.99	0.47	[0.70; 0.90]	[0.85; 1.16]	[0.62; 1.35]	0.08
Anger attribution bias^×^	R,M > P	0.22–0.76	0.49	0.10	0.52	0.08	0.47	0.12	[0.46; 0.52]	[0.50; 0.54]	[0.40; 0.55]	0.03
Hostile intent attribution^×^	R,M > P	0.00–0.69	0.26	0.19	0.33	0.20	0.27	0.26	[0.21; 0.32]	[0.25; 0.41]	[0.06; 0.48]	0.03

Study 3												
			R (*n* = 76)		M (*n* = 27)		P (*n* = 20)		Reactive	Mixed	Proactive	η_p_^2^
	*H*_*A*_	Range	*M*	*SD*	*M*	*SD*	*M*	*SD*	95% CI	95% CI	95% CI	
Teacher-report												
ADHD symptoms^×^	R,M > P	0.00–26.00	12.00^a^	6.69	15.15^ab^	6.29	16.42^b^	5.98	[10.55; 13.50]	[12.80; 17.41]	[13.73; 19.14]	0.07^*^
Emotional symptoms^×^	R,M > P	0.00–8.00	1.99	2.09	1.78	1.31	2.68	2.41	[1.55; 2.45]	[1.30; 2.28]	[1.71; 3.72]	0.02
Conduct problems^×^	P,M > R	0.00–21.00	3.17	3.64	5.52	5.64	5.74	5.72	[2.37; 4.00]	[3.55; 7.67]	[3.47; 8.30]	0.07
Peer problems^×^	R,M > P	0.00–11.00	3.50	2.57	4.67	2.39	3.79	3.38	[2.94; 4.08]	[3.83; 5.52]	[2.38; 5.34]	0.03
Psychopathy	P,M > R	0.05–1.55	0.58^a^	0.30	0.75^b^	0.29	0.83^b^	0.32	[0.51; 0.65]	[0.65; 0.86]	[0.69; 0.97]	0.10^*^
Empathy	P,M < R	-3.31–2.08	-0.51^a^	1.11	-1.40^b^	1.02	-1.68^b^	1.01	[-0.76; -0.26]	[-1.77; -1.03]	[-2.10; -1.27]	0.18^*^
Parent-report												
ADHD symptoms^×^	R,M > P	0.00–14.00	9.69	3.82	10.38	3.40	9.84	3.29	[8.80; 10.52]	[9.04; 11.64]	[8.23; 11.32]	< 0.01
Emotional symptoms^×^	R,M > P	0.00–12.00	3.80	2.74	3.96	2.58	3.68	2.21	[3.20; 4.43]	[2.96; 4.96]	[2.75; 4.63]	< 0.01
Conduct problems^×^	P,M > R	0.00–21.00	5.57	4.31	6.46	4.34	4.11	3.38	[4.69; 6.51]	[4.83; 8.12]	[2.64; 5.72]	0.03
Peer problems^×^	R,M > P	0.00–15.00	5.93	3.52	6.42	4.08	5.05	3.17	[5.17; 6.72]	[4.91; 8.15]	[3.61; 6.54]	0.02
Psychopathy	P,M > R	0.20–1.65	0.81	0.32	0.82	0.32	0.76	0.26	[0.74; 0.88]	[0.71; 0.95]	[0.64; 0.88]	< 0.01
Empathy	P,M < R	-3.87–2.47	-0.02	1.35	-0.53	1.33	-0.01	1.36	[-0.33; 0.27]	[-1.04; -0.04]	[-0.66; 0.58]	0.03
Empathic response	P,M < R	0.00–14.00	4.93	2.98	3.41	3.38	4.40	2.91	[4.28; 5.59]	[2.25; 4.66]	[3.09; 5.67]	0.04

The column *H*_*A*_ shows how the subgroups are predicted to deviate from each other. Underlined predictions were at least partly supported. Subgroups with different superscripts have non-overlapping 95% CIs

^×^There were missing scores for Study 1 Provoked anger, Hostile intent attribution (*n* = 1), and Social Acceptance (*n* = 7) because children failed to complete these measures; for Study 2 Anger attribution bias (*n* = 18) because children were absent from school at the day of testing; for Study 2 Psychopathy and Hostile intent attribution (*n* = 46) because only one of the two studies included these measures; and for Study 3 TRF (*n* = 1) and CBCL measures (*n* = 3) because reporters did not complete them

### Discussion

Study 1 supported the existence of reactive, proactive, and mixed subtypes of children. The data favored the *predominant* version of this *both subtypes hypothesis*, suggesting that children of the reactive subtype also tended to display some proactive motives, and vice versa. The distinctiveness of these subtypes received partial support from the data: 5 out of 18 measures significantly discriminated between the subtypes.

These findings emerged from a school-based community sample, limiting generalization to children with more serious aggressive behavior problems. For instance, the proactive subtype found in this sample may reflect a subgroup of relatively well-adjusted children who engage in bullying to increase their social status (Sutton et al., 1999). To test whether this model would provide the best fit for the data in samples of children with behavior problems, we attempted to replicate these findings using two clinical samples.

## Study 2: Clinical Sample #1

### Method

#### Participants

 Data were used from two studies on the effectiveness of a cognitive bias modification procedure in boys with behavior problems (Hiemstra, 2019; Hiemstra et al., 2019 [study 1]). The sample included *N* = 165 boys recruited in 2014–2015 from eight Dutch schools providing special education for children with behavior problems. In the Netherlands, children are only referred to special education if they have at least one DSM diagnosis. Schools distributed consent letters to parents or caretakers. Boys who received active consent participated in the study. This study was approved by the local ethics review board at Utrecht University, Faculty of Social and Behavioural Sciences. We excluded children with incomplete scores on reactive or proactive motives (*n* = 50). The final sample consisted of *n* = 115 boys ages 8–13 (*M*_age_ = 11.65, *SD* = 1.17). Case records were available from four of the schools (*n* = 63), and we reviewed them to provide information on the DSM diagnoses of the sample. Specifically, these 63 boys were diagnosed with Attention-Deficit Hyperactivity Disorder (ADHD; 19.0%), Oppositional Defiant Disorder or Conduct Disorder (ODD/CD; 9.5%), Autism Spectrum Disorder (ASD; 25.4%), both ADHD and ODD/CD (11.1%), both ADHD and ASD (22.2%), or another disorder (12.7%).

#### Instrument for Reactive and Proactive Aggression (IRPA)

 Teachers completed the IRPA at pre-assessment, before the intervention was implemented. The questionnaire was similar to Study 1, except for three changes. First, the aggression item about gossiping was replaced by an item about threatening others—a form of verbal aggression more prevalent in boys. Second, items concerned the past week rather than the past month. Third, teachers rated motive items for all aggressive behavior at once rather than for each aggression item separately. Internal consistency reliability was good for all subscales (aggression: α = 0.87; reactive motives: *r* = 0.37; proactive motives: *r* = 0.36). Reactive and proactive motive scales were correlated in this sample (*r* = 0.36, *p* < 0.001).

#### Subgroup Comparison Measures

 We selected measures from the data collected during the pre-assessment period. Of note, some of the measures were only used in one of the two studies and so were available only for 60% of the merged sample (see Table 3).

#### Psychopathy

 Teachers completed the Antisocial Process Screening Device (APSD; Frick & Hare, 2001). The APSD includes 20 items on impulsivity, narcissism, and callous/unemotional traits and is rated on a 3-point Likert scale from 0 (*not at all true*) to 2 (*definitely true*). Scores were averaged across items (α = 0.84).

#### Anger Attribution Bias

 Children completed a computer task presenting them with 45 images of boys’ facial expressions (Hiemstra et al., 2019). These images were created by morphing photos of 9 boys making happy and angry facial expressions into 15 different expressions for each boy, which differed in the level of ambiguity. Children were presented with one image at a time, in random order. Following each image, children indicated whether the boy in the image looked happy or angry. Anger attribution bias was calculated as the proportion of “angry” responses across the 45 trials.

#### Hostile Intent Attribution

 Children listened to an experimenter reading aloud 4 vignettes describing ambiguous peer provocations, similar to the ones used in Study 1 (De Castro et al., 2005). Children were asked two questions following each vignette: “Why did he do that?” and “How did he intend it?” Open-ended responses to the first question were coded by two independent coders as *benevolent, accidental, ambiguous, hostile,* or *irrelevant* (ICC = 0.94). We assigned a 1 (*hostile*) if both coders assigned this code and 0 (*nonhostile*) otherwise. Children responded to the second question by choosing between *mean, a little mean, accidental, a little nice,* or *nice*. We assigned a 0 (*nonhostile*) if they selected *accidental, a little nice,* or *nice*, 1 (*hostile*) if they selected *mean* and 0.5 if they selected *a little mean*. Scores were averaged across questions and vignettes (α = 0.53).

#### Analytical Approach

 We used the same analytical approach as in Study 1, with the exception of how we accounted for clustering within classroom. Specifically, we used complex sampling (Asparouhov, 2005) rather than multi-level modelling; we were unable to use multi-level modeling because the smaller sample size led to nonidentification issues.

### Results

We first tested which hypothesis best fit the data of this clinical sample. We planned to select the model with the lowest AIC, BIC, and aBIC, and sufficient entropy (i.e., > 0.80). However, results for the fit indices were inconsistent. The *reactive only – predominant model* had the lowest BIC, but the second-lowest AIC and aBIC, and insufficient entropy (i.e., below 0.80; Table 2). Hence, we selected the *both subtypes – predominant model*, which had the lowest AIC and aBIC, and an entropy value that almost reached 0.80. These results replicated Study 1, in that the best-fitting model was the *both subtypes – predominant model.* However, the pattern of findings was not as consistent as those that emerged in Study 1.

Next, we applied the constraints of the *both subtypes – predominant model* to create subgroups in this clinical sample. We found that 55.7% of children were classified as predominantly reactive, with higher scores on reactive motives (*M* = 1.94, *SD* = 0.94) versus proactive motives (*M* = 0.53, *SD* = 0.54). Another 10.4% of children were classified as predominantly proactive, with higher scores on proactive motives (*M* = 1.17, *SD* = 0.87) versus reactive motives (*M* = 0.14, *SD* = 0.26). The remaining 33.9% of children were classified as mixed, with similar scores on reactive motives (*M* = 1.88, *SD* = 0.84) and proactive motives (*M* = 1.59, *SD* = 0.66). As in Study 1, we found a proactive subgroup, even though reactive motives were on average more prevalent (*M* = 1.73, *SD* = 1.01) than proactive motives (*M* = 0.95, *SD* = 0.79). In this Study, the mean frequency of aggression did not significantly differ between the mixed (*M* = 1.33, *SD* = 0.80, CI[1.09; 1.58]), reactive (*M* = 0.95, *SD* = 0.76, CI[0.78; 1.14]), and proactive subgroups (*M* = 0.82, *SD* = 0.86, CI[0.38; 1.30]), as evidenced by overlapping bootstrap 95% confidence intervals for all subgroups.

Last, we compared the subgroups on the three measures available for this sample. Table 3 includes these measures, the hypothesized effect for each measure, and the descriptive statistics on each measure for children in the predominantly reactive, predominantly proactive, and mixed subgroups. There were not any significant differences between the subgroups, as indicated by overlap between all confidence intervals.

### Discussion

The findings from Study 2 replicated Study 1, in that they supported the *both subtypes – predominant model,* although support was not as strong due to the lack of convergence across fit statistics*.* The distinctiveness of the proactive and reactive subgroups was not supported. However, analyses were hindered by low psychometric quality of the hostile intent attribution measure and low statistical power for most measures (i.e., *n* = 67). Therefore, we turned a second clinical sample from a data set with a greater range of measures that would also test the *both subtypes – predominant model* in a younger sample (i.e., ages 6–8).

## Study 3: Clinical Sample #2

### Method

#### Participants

 Data were used from a study of empathy in children with developmental disorders (Deschamps et al., 2014, 2015, 2018). Participants were *N* = 138 children recruited in 2009–2011 from an outpatient psychiatry clinic in a Dutch city. A clinician approached parents of all children whose DSM diagnosis was confirmed using the parent version of the Diagnostic Interview Schedule for Children (DISC, module E; Shaffer et al., 2000). Children whose parents gave written informed consent participated in the study. This study was approved by the Medical Ethics Committee of the University Medical Center Utrecht. We excluded children with incomplete scores on reactive and proactive motives (*n* = 15). The final sample consisted of *n* = 123 children ages 6–8 (78.0% boys; *M*_age_ = 6.87, *SD* = 0.60). Children had ADHD (28.5%), ODD/CD (15.4%), or both ADHD and ODD/CD (56.1%).

#### Instrument for Reactive and Proactive Aggression (IRPA)

 Teachers completed the IRPA following the procedures of Study 1. The IRPA has been used in previous samples of 6-year-olds, supporting its reliability and enhanced ability to discriminate between reactive and proactive motives, as indicated by weak correlations between the two scales (e.g., Austin et al., 2017; Van Dijk et al., 2017). In our sample, internal consistency reliability was good for all subscales (aggression: α = 0.77; reactive motives: *r* = 0.53; proactive motives: *r* = 0.31). Reactive and proactive motives were not significantly correlated (*r* = 0.12, *p* = 0.206).

#### Subgroup Comparison Measures

 Where possible, we selected measures and constructs similar to those used in Study 1.

#### Symptoms: ADHD Symptoms, Emotional Symptoms, Conduct Symptoms, and Peer Problems

 Parents completed the Child Behavior Checklist 6–18 (CBCL), and teachers completed the Teacher Report Form (TRF) (Achenbach & Rescorla, 2001). We assessed ADHD symptoms using the ADHD syndrome scale, emotional symptoms using the anxious-depressive symptom scale, conduct symptoms using the CD syndrome scale, and peer problems using the social problems scale.

#### Psychopathy

 Parents and teachers completed the Antisocial Process Screening Device (APSD; Frick & Hare, 2001; see Study 2 for details). Reliability in this sample was good for both parent-report (α = 0.81) and teacher-report (α = 0.82).

#### Empathy

 Parents and teachers completed the Griffith Empathy Measure (GEM; Dadds et al., 2008). The measure was adapted to assess empathy in response to sadness or distress (Deschamps et al., 2018). This approach resulted in a 15-item scale for parents and a 13-item scale for teachers. Items were rated on a scale from -4 (*strongly disagree*) to + 4 (*strongly agree*). Reliability was good for both parent- (α = 0.87) and teacher-report (α = 0.85).

#### Empathy-induced Responding

 Children completed the Interpersonal Response Task, a computer-based task assessing prosocial responses to sadness and distress (Dadds & Hawes, 2004; also see: Deschamps et al., 2015). The task consists of a ball-throwing game in which children can choose to throw the ball to another player, who displays a progressively sadder facial expression upon not receiving the ball (and the concomitant monetary reward). Empathy-induced responding is assessed as the number of trials out of 20 in which children decide to throw the ball to the “sad” player.

#### Analytical Approach

 We used the same analytical approach as in Study 1 and 2. There was no need to account for clustering as children were recruited through one clinic.

### Results

We first tested which hypothesis best fit the data of this sample. The LPA showed that the *pure* models did not fit the data at all; entropy indicated that no children were classified as purely reactive. Results for the fit indices were inconsistent. Of the two *predominant* models, the *reactive only model* had the lowest BIC, but the *both subtypes model* had the lowest AIC and aBIC, and the highest entropy (Table 2). We therefore selected the *both subtypes model*.

We created the subgroups by applying the constraints the selected model. We found that 61.8% of children were classified as predominantly reactive, with higher scores on reactive motives (*M* = 1.81, *SD* = 0.87) versus proactive motives (*M* = 0.45, *SD* = 0.46). Another 16.3% of children were classified as predominantly proactive, with higher scores on proactive motives (*M* = 1.31, *SD* = 0.82) versus reactive motives (*M* = 0.43, *SD* = 0.51). The remaining 22.0% were classified as mixed, with similar scores on reactive motives (*M* = 1.49, *SD* = 0.67) and proactive motives (*M* = 1.34, *SD* = 0.53). As in the previous studies, we found a proactive subgroup, despite the higher prevalence of reactive motives (*M* = 1.51, *SD* = 0.92) versus proactive motives (*M* = 0.78, *SD* = 0.78). The mean frequency of aggression did not differ between the mixed (*M* = 1.10, *SD* = 0.91, CI[0.79; 1.44]), reactive (*M* = 0.73, *SD* = 0.43, CI[0.64; 0.83]), and proactive subgroups (*M* = 0.60, *SD* = 0.50, CI[0.40; 0.83]).

Last, we compared these subgroups on our selected measures. Table 3 includes these measures, the hypothesized effect for each measure, and the descriptive statistics on each measure for children in the predominantly reactive, predominantly proactive, and mixed subgroups. A MANOVA revealed an overall effect of subgroup across these measures, *F*(26, 212) = 1.91, *p* = 0.007, η_p_^2^ = 0.19. The bootstrap 95% confidence intervals (Table 3) indicate that hypotheses were at least partially supported for teacher-reported psychopathy^3^ and empathy, but not for the remaining constructs. Thus, these data provide limited support for the distinctiveness of the subgroups emerging from the *both subtypes – predominant model*.

### Discussion

Findings from Study 3 replicated Study 1 and 2, in that the strongest support emerged for the *both subtypes – predominant model*. Only modest support emerged for the distinctiveness of the groups, with 2 out of 13 measures discriminating between them.

## General Discussion

The present study examined whether there are distinct groups of children with reactive versus proactive motives for their aggressive behavior. We used confirmatory LPA to test two competing hypotheses arising from the literature, the *reactive only hypothesis* versus the *both subtypes hypothesis*. Both hypotheses predict reactive and mixed subtypes, but only the *both subtypes hypothesis* predicts an additional proactive subtype. We tested these hypotheses on existing data from three middle childhood samples to test these hypotheses: a community sample of children displaying aggression and two clinical samples of children with aggressive behavior problems, providing the opportunity to evaluate the robustness of our findings across samples.

### Both Reactive and Proactive Subtypes

Results converged across samples to suggest that the *both subtypes hypothesis* best described the data, supporting the existence of reactive, mixed, and proactive subtypes of aggressive children. This hypothesis was supported across community and clinical samples and across ages (i.e., from 6 to 13 years), suggesting that children have developed distinct patterns of reactive and proactive aggression by the time they enter primary school. These findings provide the most robust evidence to date for the existence of both reactive and proactive subtypes of aggressive children.

We further tested both *pure* and *predominant* versions of the hypotheses. The *pure* version predicted that children’s aggression is fully motivated by one motive and not the other; this model was not supported. Instead, the *predominant* version of the *both subtypes model* was supported, suggesting that children from both the reactive and proactive subtypes also evidenced the other motive, although to a lesser degree. These findings resonate with scholarly work on the development of reactive and proactive aggression, suggesting that young children aggress for primarily reactive reasons, with some children developing proactive motives as well as they age and learn that aggression can result in desired outcomes (De Castro, 2004; Dodge et al., 1997). Of course, our findings do not rule out the possibility that a few children aggress entirely for one motive or the other, such as children exhibiting severe conduct problems (Frick et al., 2014). However, these children may be so rare that inferential statistics in small-to-medium samples such as ours do not identify them. Future research using larger samples may provide a more thorough answer to this question.

Previous studies using person-based analyses supported only reactive and mixed subtypes of aggressive youth (Colins, 2016; Cui et al., 2016; Euler et al., 2017; Marsee et al., 2014; Muñoz et al., 2008; Pang et al., 2013; Smeets et al., 2017; Thomson & Centifanti, 2018). These findings suggest that proactive aggression may only occur in children who also display reactive aggression, should thus be seen as a severity marker rather than a distinct subtype, and may bear little relevance for distinguishing subtypes of aggressive children (Marsee et al., 2014; Smeets et al., 2017). In contrast, the present study did reveal a group of 10–24% of children who display predominantly proactive aggression, which we detected likely because of our use of a questionnaire with improved ability to discriminate between reactive and proactive motives (i.e., yielding low rather than high intercorrelations; Polman et al., 2009). If replicated, these findings have both theoretical and clinical implications. Theoretically, they suggest that proactive aggression may be a distinct subtype, rather than a mere marker of aggression severity. Clinically, they suggest that it may prove effective to tailor separate interventions to reactive and proactive subtypes of aggressive children.

### Distinctiveness of the Subtypes

A secondary goal of the study was to compare the reactive, mixed, and proactive subtypes on variables that previous literature suggests uniquely correlate with reactive and proactive aggression. Support for the distinctiveness of the subtypes was limited. Across the three samples, only 7 of 34 measures discriminated between the reactive, mixed and proactive subtypes in the expected direction. In line with previous research, the reactive subtype showed higher levels of emotional symptoms, victimization, and empathy and lower levels of conduct problems, psychopathy, bullying, and coercive strategy use compared to the proactive and mixed subtypes (for reviews, see: Hubbard et al., 2010; Merk et al., 2005), providing partial support for the reactive and proactive subtypes identified in our samples.

However, the subtypes did not differ on many other variables, including ADHD symptoms, social information processing measures, and several peer relations measures. Lack of power may partially explain these null findings, in that mean differences between the subtypes were in expected directions for teacher-rated psychopathy in Study 2 and teacher-rated conduct problems in Study 3. However, many other variables did not follow predicted patterns. One possible explanation for this discrepancy is that children’s reactive or proactive aggression is specific to the school context. We based our subtypes on teacher-report and also tended to find subtype differences for reports by classmates and teachers (i.e., 7 out of 17 measures), but not by parents or children themselves (i.e., 0 out of 17 measures). For this reason, future research may investigate the stability of the reactive and proactive subtypes across contexts. In any case, our inconsistent validation results warrant replication in larger samples to further investigate the existence of distinct reactive and proactive subtypes.

One finding from our subgroup analyses stands out as particularly in contrast to hypotheses and previous literature. That is, teachers reported less ADHD symptoms for children of the reactive subtype versus the mixed (Study 1) and proactive subtypes (Study 3). This finding contrasts with research linking reactive aggression to inattention and impulsivity problems (Merk et al., 2005), but aligns with work showing that hyperactivity at age 7 predicts future proactive aggression (Raine et al., 2006). Perhaps, our ADHD measure mostly captured variance in hyperactivity, as this may be the most visible symptom for teachers. Future research may clarify this issue by using separate assessments of the inattention, impulsivity, and hyperactivity dimensions of ADHD.

### Strengths and Limitations of the Study

The present study had several strengths, each of which also presented limitations. First, by using the IRPA, we disentangled children’s motives for aggression from the forms of that aggression. This resulted in much lower correlations between reactive and proactive motives than are found in previous studies and, consequently, increased power to detect distinct reactive and proactive subtypes of aggressive children. However, as the IRPA assesses children’s motives independent from the frequency or severity of their aggression, it does not capture the frequency or severity of *reactive* or *proactive aggression* (i.e., as one construct). Thus, the children in our subtype groups may have varied widely in the frequency or severity of their reactive or proactive aggression. Moreover, the proactive subscale of the IRPA assesses dominance and intimidation, but lacks items on the instrumental dimension of proactive aggression (Polman et al., 2009). These limitations potentially reduced the power to detect subgroup differences on our social-emotional measures.

Second, we used a confirmatory LPA approach. As the literature revealed competing hypotheses on the existence of particularly a proactive subtype, we had strong theoretical reasons to define competing models. Confirmatory LPA provides a more direct test of these hypotheses than exploratory LPA, as it not only sets the number of classes but also specifies how these classes should differ (Finch & Bronk, 2011). Specifically, we were able to specify within-class constraints (e.g., that scores within one class should be higher on reactive versus proactive motives) whereas exploratory LPA automatically uses between-class comparisons (e.g., that scores on reactive motives should be higher for one class versus another). Yet, as previous person-based studies used exploratory LPA, the question arises whether our novel detection of a predominantly proactive group may result from our analytical approach, rather than our use of the IRPA, as we have argued throughout this paper. We therefore re-analyzed all data using exploratory LPA. Results revealed a proactive subgroup in all three studies, thus supporting the *both subtypes hypothesis* (Appendix S4, Supplementary Information).

Another potential limitation of confirmatory LPA is that it required us to pre-define the exact model constraints. Such a priori choices may seem arbitrary. One choice we made was to analyze raw scores of reactive and proactive motives, so that the created subgroups reflected teachers’ judgments of whether children used reactive or proactive motives more frequently (or about equally, for the mixed subtype). This approach, however, ignores that reactive motives were more prevalent than proactive motives. We therefore reran all analyses using standardized scores. Findings revealed a predominantly proactive subgroup in all three studies, again supporting the *both subtypes hypothesis* (Appendix S2, Supplementary Information). Another choice we made is how we defined the subgroups (e.g., we defined the predominant reactive subtype as scoring at least 1.5 times higher on reactive versus proactive motives). We therefore conducted sensitivity analyses for a range of specifications (i.e., ratios of 1.25, 1.75 and 2.00), both using raw scores and standardized scores (Appendix S1 and S3 in the Supplementary Information, respectively). Again, all analyses detected a predominantly proactive subgroup, providing robust support for the *both subtypes hypothesis*.

Third, we used data from three samples, enabling us to test the robustness of the *both subtypes hypothesis* across community and clinical samples and across ages (i.e., 6–13). Using existing data, though, resulted in several limitations. First, our sample sizes were suboptimal to detect subtype differences on our social-emotional measures, especially for Studies 2 and 3. Moreover, we selected measures from the data available, rather than picking measures most relevant to identify reactive and proactive subtypes (e.g., physiological measures), or precise enough to do so (e.g., differentiating between conduct problems with and without callous-unemotional traits; Frick et al., 2003). Finally, our findings are limited to middle childhood samples. Although it seems plausible that the subtypes found in our samples would also be present in older samples—after all, reactive and proactive aggression are thought to become more differentiated over time (Vitaro et al., 2006)—work with adolescent samples (and especially longitudinal work) is needed to test this hypothesis.

## Conclusion

This study suggests that distinct subtypes of aggressive children with predominantly reactive, proactive, and mixed motives may exist. If future work replicates our findings, the implications for both research and clinical practice may be substantial. For research, our findings suggest that the choice of questionnaire to assess reactive and proactive motives for aggression may play an important role in the results that emerge. Moreover, the identification of separate reactive and proactive subtypes may advance our understanding of differential etiological factors predicting which children will go on to develop reactive aggression, proactive aggression, or both (e.g., difficult temperament versus harsh parenting; Vitaro et al., 2006), as well as the disparate outcomes that these groups of children may display (e.g., poor versus average academic performance; Fite et al., 2013; dating violence versus delinquency; Brendgen et al., 2001). For clinical practice, these findings suggest that it may be important to tailor treatment to children’s individual needs, for instance by targeting emotion regulation problems in predominantly reactive children versus response decision processes in predominantly proactive children (Hubbard et al., 2010). We hope our work will inspire further research on the distinctiveness, etiology, and treatment of children displaying reactive versus proactive aggression.

## Supplementary Information

Below is the link to the electronic supplementary material.

Supplementary file1 (PDF 540 KB)

## Data Availability

The data that support the findings of this study are available through the Open Science Framework at https://osf.io/7weub.

## Footnotes

1 We thoroughly discussed the appropriate criterion. We could not use the customary cut-off of one standard deviation from the mean because we needed to define the ratio between motives *within* children. We chose a ratio of 1.5 because a ratio close to 1 would yield reactive and proactive subtypes similar to the mixed subtype, whereas a ratio close to 2 would yield too stringent of a test of the hypothesis, resembling the *pure* hypotheses.

2 We calculated reliability for the motive scales using the mean inter-item correlation, which does not depend on the number of items as does Cronbach’s alpha. Reliability was sufficient (i.e., r ≥ .15; Clark & Watson, 1995).

3 We explored possible differences between the two psychopathy subscales *callous-unemotional (CU) traits* and *impulsivity*, expecting that CU traits may be higher in the proactive and mixed subgroups versus the reactive subgroup. For Study 3, results supported this expectation, suggesting that the obtained effect for psychopathy is driven by differences in children’s CU traits (for details, see Appendix S6 in the Supplementary Information).

## Code Availability

The syntax of the analyses run for this study are available through the Open Science Framework (see link above).
