# Supplementary material for: Do Distinct Groups of Reactively and Proactively Aggressive Children Exist? A Confirmatory Latent Profile Approach
Source: Res Child Adolesc Psychopathol. 2021 Apr 21;49(10):1303–17. doi: 10.1007/s10802-021-00813-0 (PMC8380234; doi:10.1007/s10802-021-00813-0)
Supplement: Supplementary file 1 — Supplementary file1 (PDF 540 KB) [file 10802_2021_813_MOESM1_ESM.docx]

**Do Distinct Groups of Reactively and Proactively Aggressive Children Exist?**

**A Confirmatory Latent Profile Approach**

**Supplementary Information**

Anouk van Dijk^12^, Julie A. Hubbard^3^, Peter K. H. Deschamps^4^, Wieteke Hiemstra^1^, and Hanneke Polman^1^

^1^ Department of Psychology, Utrecht University, Utrecht, The Netherlands

^2^ Research Institute of Child Development and Education, University of Amsterdam, Amsterdam, The Netherlands

^3^ Department of Psychological and Brain Sciences, University of Delaware, Newark, Delaware, United States of America

^4^ Department of Psychiatry, University Medical Center Utrecht, Utrecht, The Netherlands

The data that support the findings of this study are available through the Open Science Framework at <https://osf.io/7weub/?view_only=0d225081904643c8a8425526d61cedd2>.

**Overview of Appendices S1-6**

**Sensitivity Analyses**

We reanalyzed the Results sections of Studies 1-3 in a number of different ways. First, we reanalyzed the data using a variety of different ratios to classify children into groups (1.25, 1.75, and 2.00, in addition to the 1.50 ratio reported in the manuscript). Second, we reanalyzed the confirmatory Latent Profile Analysis (LPA) using standardized scores as opposed to the raw score analyses reported in the manuscript, and repeated this analysis for each ratio. Finally, we reanalyzed the data using exploratory LPA rather than confirmatory LPA, given that exploratory LPA is more prevalent in the literature. For an overview of all sensitivity analyses, please see the diagram below:


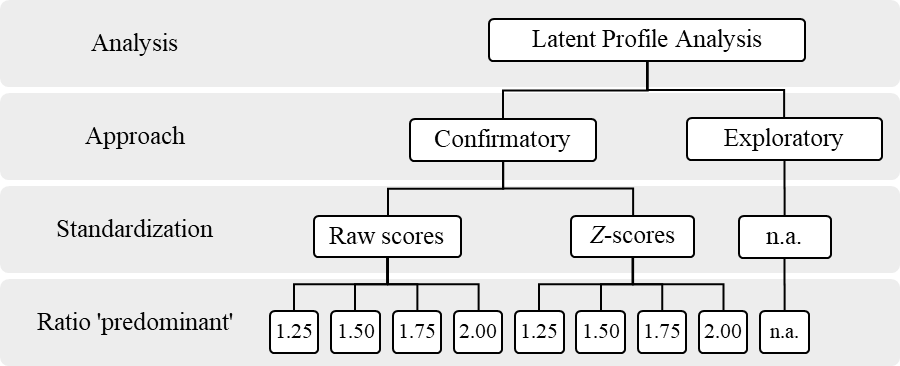


In paper S2 S4 S1 S3

Please note that the elements of standardization and ratio to define predominant only pertain to confirmatory LPA, which uses pre-defined ratios of within-person reactive versus proactive motives. These elements do not affect exploratory LPA, which draws from between-person variance in reactive and proactive motives to create subgroups.

**Additional Analyses**

We also conducted additional analyses for test for gender differences in our obtained subgroups, and to test whether subgroup comparison findings for psychopathy would differ between the subscales of CU-traits and impulsivity:

- Appendix S5: Gender Differences
- Appendix S6: Subgroup Comparison Analyses for the Psychopathy Subscales

**Appendix S1: Sensitivity Analyses Using Different Ratios to Define ‘Predominant’**

We reran all analyses originally conducted using confirmatory LPA, raw scores, and a ratio of 1.50, but now using ratios of 1.25, 1.75 and 2.00 to define the predominantly reactive and proactive subgroups.

**Latent Profile Analyses.** Ratio had little effect on our findings (Table S1). Results were the same for all ratios in Study 1 and 3, and highly similar in Study 2. In Study 1, all ratios yielded the same conclusion: all fit statistics supported the *both subtypes model*. Similarly, in Study 3 all ratios yielded the same conclusion: BIC supported the *reactive only model*, but AIC, aBIC and entropy supported the *both subtypes model*. In Study 2, we found one difference between ratios: AIC supported the *both subtypes model* for ratios 1.25 and 1.50, but the *reactive only model* for ratios 1.75 and 2.00. The other fit statistics (i.e., BIC, aBIC and entropy) yielded the same conclusion for all ratios. Overall, it seems that variations in how we defined “predominant” had little influence on the fit of our models.

We did find one ratio effect for AIC in Study 2. How should we interpret that? This effect is most likely caused by the small sample size of that study. In fact, the “lost” support of AIC for higher ratios in Study 2 resembles a general trend in our sensitivity analyses for all studies, namely, that statistical fit (i.e., AIC, BIC and aBIC) decreases with higher ratios, whereas classification quality (i.e., entropy) increases with higher ratios. This pattern indicates that more extreme definitions yield clearer delineations between the subgroups (i.e., higher entropy), but decrease the sizes of the reactive and proactive subgroups, thereby worsening fit for the overall dataset. Because Study 2 already had a small sample size, with only *n* = 12 children classified as proactive in our original 1.50-ratio analyses, it is only logical that further curtailing this subgroup worsened overall fit. We thus interpret the “lost” support of AIC as a sample-size-related issue rather than a substantive finding—an interpretation that resonates with the absence of ratio effects for all other fit statistics in Studies 1, 2, and 3.

**Calculating the Subgroups.** We next created subgroups for the *both subtypes – predominant model* for each of the ratios of 1.25, 1.50, 1.75, and 2.00. In Study 1, for all ratios, group sizes were largest for the reactive subgroup (*n*s = 142, 132, 130, and 122, resp.), with lower sizes of the mixed (i.e., *n*s = 23, 41, 44, 58) and proactive subgroups (*n*s = 63, 55, 54, and 48). We found the same pattern of group sizes in Study 2 (reactive *n*s = 81, 64, 62, 47; mixed *n*s = 19, 39, 41, 57; proactive *n*s = 15, 12, 12, 11) and Study 3 (reactive *n*s = 85, 76, 70, 62; mixed *n*s = 16, 27, 34, 48; proactive *n*s = 22, 20, 19, 13). Logically, sizes of the mixed subgroup increase with stricter ratios, whereas sizes of the other subgroups decrease.

**Subgroup Comparison Analyses.** The subgroup comparison analyses yielded the same conclusions as to whether our expected group differences were supported by the data regardless of ratio, except for one measure in Study 3 (Table S2). In Study 3, using a 1.50 ratio, we initially found no significant differences for teacher-rated psychopathy, even though mean patterns suggested that children in the mixed subgroup had higher scores than children in the reactive subgroup. The sensitivity analyses revealed that this difference was significant for all ratios except for the 1.50 ratio reported in the paper.

**Conclusion.** Overall, the sensitivity analyses for ratio effects revealed highly similar results using different ratios, suggesting that our choice of ratio for our confirmatory models had little effects on our findings.

**Appendix S2: Sensitivity Analyses Using Standardized Scores**

We found that proactive motives were less prevalent than reactive motives (i.e., *M* = 0.61 vs. *M* = 1.11 in Study 1, *M* = 0.95 vs. *M* = 1.73 in Study 2, and *M* = 0.78 vs. *M* = 1.51 in Study 3). We therefore explored whether our findings would change if we controlled for this lower prevalence by using standardized scores. Specifically, we reran all analyses, this time using *z*-scores of reactive and proactive motives as input for our analyses.

**Latent Profile Analyses.** Standardization affected our findings in Study 1, but not Study 2 and 3 (Table S3). In Study 1, BIC and entropy supported the *both subtypes – predominant model* using raw scores, but favored the *reactive only – predominant model* using standardized scores. AIC and aBIC favored the *both subtypes – predominant model* using either raw or standardized scores. We continued with this model for all three studies throughout the remainder of Appendix S2 to enhance comparability between these sensitivity analyses and our main findings.

**Calculating the Subgroups.** We next created subgroups for the *both subtypes – predominant model* based on the standardized scores. In Study 1, we found that 47.4% of children were classified as reactive, 49.6% as proactive, and only 3.1% as mixed (vs. 57.9%, 24.1%, and 18.0% using raw scores). In Study 2, we found 40.0% reactive, 50.4% proactive, and 9.6% mixed (vs. 55.7%, 10.4% and 33.9%). And in Study 3, we found 40.7% reactive, 50.4% proactive, and 8.9% mixed (vs. 61.8%, 16.3% and 22.0%). This pattern of findings suggests that the better fit of the 2-class model in Study 1 for BIC and entropy was driven not by the lack of a proactive subgroup, but by the lack of a mixed subgroup. Thus, the standardized findings still support the *both subtypes hypothesis* for all three studies.

Why did the mixed subgroup decrease upon standardization? We think this pattern is caused by a classification shift from mixed to proactive for those children with similar frequencies of reactive and proactive motives according to their teachers. Using raw scores, these children were classified as “mixed”—after all, they had similar scores on proactive and reactive motives, because their teachers viewed them as using both motives for aggression with similar frequency. Using *z*-scores, however, these children were classified as “proactive” because they now had higher scores on proactive versus reactive motives, because proactive motives were less prevalent. Apparently, there were few children who had average levels of both reactive and proactive motives, resulting in quite small mixed subgroups across studies when scores were standardized.

**Subgroup Comparison Analyses.** The comparison of *z*-score-based subgroups yielded the same conclusions as to whether our expected group differences were supported by the data with two exceptions. Specifically, support was lost for coercive strategy use in Study 1 and teacher-rated psychopathy in Study 3 (Table S4) using standardized scores in place of raw scores. For these two measures, the significant group differences disappeared, perhaps because many children previously classified as “mixed” now were in the proactive group, thus reducing the contrast between the subgroups.

**Conclusion.** The sensitivity analyses for standardization yielded similar results in that they reveal a proactive subgroup, supporting the *both subtypes hypothesis* and resonating with the findings reported in our paper. However, they also reveal that standardization strongly affects the interpretation of the subgroups. Children with similar absolute levels of reactive and proactive motives according to their teachers were seen as “mixed” using the raw score approach, but as “proactive” using the standardized approach. This gives rise to a substantive question: Should we classify children as proactive for having above-average proactive motives, even if their absolute levels of reactive motives are higher (i.e., use *z*-scores)? Or should we classify children as proactive for displaying proactive motives more often than reactive motives (i.e., use raw scores)? Both options are defensible. We retained the raw-score analyses in the paper and report the results for the standardized scores here.

**Appendix S3: Sensitivity Analyses Using Standardized Scores *and* Different Ratios to Define ‘Predominant’**

To be thorough, we also examined ratio effects for the analyses based on standardized scores. We reran all analyses conducted in Appendix S2, this time using ratios of 1.25, 1.75 and 2.00 to define the predominantly reactive and proactive subgroups.

**Latent Profile Analyses.** Ratio had no effect on our analyses using standardized scores for Study 1 and 2, but it did for Study 3 (Table S5). In Study 3, ratios 1.25 and 1.50 yielded support for the *both subtypes – predominant model*, whereas ratios 1.75 and 2.00 supported the *reactive only – predominant model*. Since our findings reported in the paper are based on the *both subtypes – predominant model*, we continued with this model for Study 3 throughout the remainder of Appendix S3 to enhance comparability between these sensitivity analyses and our main findings.

**Calculating the Subgroups.** We next created subgroups for the *both subtypes – predominant model* for each of the ratios of 1.25, 1.50, 1.75, and 2.00 based on standardized scores. In Study 1, for all ratios, group sizes were largest for the reactive subgroup (*n*s = 115, 108, 93, and 88, resp.), followed by the proactive subgroup (*n*s = 109, 113, 125, 122), and smallest for the mixed subgroup (i.e., *n*s = 4, 7, 10, 18). We found the same pattern of group sizes in Study 2 (reactive *n*s = 48, 46, 41, 38; proactive *n*s = 62, 58, 61, 55; mixed *n*s = 5, 11, 13, 22) and Study 3 (reactive *n*s = 54, 50, 46, 44; proactive *n*s = 63, 62, 64, 63; mixed *n*s = 6, 11, 13, 16).

**Subgroup Comparison Analyses.** The comparison of subgroups based on standardized scores and different ratios yielded the same conclusions for most measures as analyses reported in the paper regarding whether expected group differences were supported by the data (i.e., for 28 out of 34 measures; see Table S4). The newly added ratios revealed some additional support for our expectations. Specifically, expectations were supported in Study 1 for peer problems (ratios 1.75 and 2.00) and popularity (ratio 1.25), in Study 2 for psychopathy (ratio 1.75), and in Study 3 for parent-reported empathy (ratios 1.25, 1.75, and 2.00). One expectation that was initially supported in our main analyses became non-significant: coercive strategy use in Study 1 was supported only for a ratio of 2.00.

**Conclusion.** Overall, these sensitivity analyses revealed limited effects of the choice of ratio on our findings. The LPA support for the *both subtypes model* in Study 3 was weaker than in our analyses using raw scores, most likely because the mixed subgroup shrank considerably upon standardization, making the 2-class model more probable. Notably, results of all three studies still supported the existence of a proactive subgroup (rather, they cast doubt on the existence of a mixed subgroup). Last, the subgroup comparison analyses suggested some possible extra support for our hypotheses, but since this support was not consistent, further research is needed to verify.

**Appendix S4: Sensitivity Analyses Using Exploratory LPA**

Our study was the first to use *confirmatory* Latent Profile Analysis (LPA) to examine subgroups of reactive and proactive children. Previous studies have used exploratory LPA, testing how many classes best fit the data, and then examining the mean scores on reactive and proactive motives to describe the subgroups. To examine whether our new finding (i.e., the detection of a predominantly proactive subgroup) was caused by our analytical approach, we reanalyzed our data using exploratory LPA and testing fit of the 1- to 4-class models.

**Latent Profile Analyses.** In Study 1, the 4-class model best fitted the data (Table S6). This model suggests there is one “reactive” subgroup (53.5% of children) with higher scores on reactive motives (*M* = 1.15, *SD* = 0.72) versus proactive motives (*M* = 0.09, *SD* = 0.14), one “mixed” subgroup (24.1%) with similar scores on reactive motives (*M* = 0.99, *SD* = 0.98) and proactive motives (*M* = 0.80, *SD* = 0.18), one “mildly proactive” subgroup (16.7%) with somewhat higher scores on proactive motives (*M* = 1.44, *SD* = 0.18) versus reactive motives (*M* = 1.01, *SD* = 0.90), and one “strongly proactive” subgroup (5.7%) with substantially higher scores on proactive motives (*M* = 2.20, *SD* = 0.27) versus reactive motives (*M* = 1.44, *SD* = 0.80). These findings support the *both subtypes hypothesis*, in that both reactive and proactive subtypes were identified in the data.

In Study 2, the fit indices did not provide a clear-cut solution (Table S6). We selected the 3-class model because it was the only one with sufficient entropy and because it had the second-best fit in terms of AIC, BIC, and aBIC. This model suggests there is one “reactive” subgroup (53.0% of children) with higher scores on reactive motives (*M* = 2.53, *SD* = 0.55) versus proactive motives (*M* = 1.31, *SD* = 0.74), one “mixed” subgroup (46.1%) with similar scores on reactive motives (*M* = 0.84, *SD* = 0.55) and proactive motives (*M* = 0.50, *SD* = 0.51), and one “proactive” subgroup of *n* = 1 child (0.8%) with higher scores on proactive motives (score = 3.33) than reactive motives (score = 0). These findings do not provide a clear answer to the research question. Although the 3-class model supports the *both subtypes hypothesis*, the proactive subgroup consisted of only one child, warranting replication.

In Study 3, the 4-class model best fitted the data (Table S6). This model suggests there is one “mildly reactive” subgroup (40.7% of children) with somewhat higher scores on reactive motives (*M* = 1.38, *SD* = 0.91) versus proactive motives (*M* = 0.82, *SD* = 0.21), one “strongly reactive” subgroup (36.6%) with substantially higher scores on reactive motives (*M* = 1.45, *SD* = 0.88) versus proactive motives (*M* = 0.11, *SD* = 0.15), one “mixed” subgroup (18.7%) with similar scores on reactive motives (*M* = 1.93, *SD* = 0.97) and proactive motives (*M* = 1.60, *SD* = 0.24), and one “proactive” subgroup (4.1%) with higher scores on proactive motives (*M* = 2.67, *SD* = 0.30) versus reactive motives (*M* = 1.47, *SD* = 0.87). These findings support the *both subtypes hypothesis*, in that both reactive and proactive subtypes were identified in the data.

**Subgroup Comparison Analyses.** We reran the comparison analyses based on exploratory LPA-based versus confirmatory LPA-based subgroups. Results yielded the same conclusion for most measures as to whether the data supported our expected group differences (i.e., for 30 out of 34 measures; see Table S7). With the exploratory LPA-based subgroups, we found additional support for two measures (i.e., parent-reported empathy and teacher-rated conduct problems in Study 3) but also lost support for two measures (i.e., emotional symptoms and victimization in Study 1). All other measures yielded the same conclusions.

**Conclusion.** The exploratory LPA replicated the confirmatory findings in that a proactive subgroup was identified in all three studies (although the Study 2 “subgroup” consisted of only one child). The distinctiveness of the subgroups on our comparison measures was similar to the confirmatory findings, with 7 out of 34 expectations supported.

**Appendix S5: Gender Differences**

We examined gender differences in the predominantly reactive, proactive, and mixed subgroups obtained from our Latent Profile Analyses. Previous person-based studies are discrepant regarding whether boys were overrepresented in the proactive and mixed subgroups (i.e., Pang et al., 2013) or not (i.e., Euler et al., 2017; Smeets et al., 2017; Thomson & Centifanti, 2018). Hence, we explored the gender composition of our subgroups in Study 1 and 3 (Study 2 involved only boys), hoping that our use of a questionnaire with improved ability to discriminate between reactive and proactive motives would shed light on this issue.

We used a χ^2^ test to examine gender differences. In Study 1 (*n* = 228, 54% boys), the subgroups did not differ in gender composition, χ^2^(2) = .07, *p* = .967, with 46.2% girls in the reactive subgroup, 45.5% in the proactive subgroup, and 43.9% in the mixed subgroup. The same result was found in our Study 3 clinical sample (*n* = 123, 78% boys): the subgroups did not differ in gender composition, χ^2^(2) = 2.73, *p* = .256, with 17.1% girls in the reactive subgroup, 30.0% in the proactive subgroup, and 29.6% in the mixed subgroup.

Our findings diverge from previous research suggesting that boys display higher levels of proactive aggression than girls (Euler et al., 2017), and may thus be overrepresented in mixed and proactive subtypes (Pang et al., 2013). This may be explained by our analytical approach. In contrast to standard LPA, we classified children according to their own ratio of reactive versus proactive motives, not their rank among other children. Our findings tentatively suggest that boys and girls may differ in their mean levels of proactive aggression (as found by previous studies), but not in their relative use of reactive versus proactive motives (as tested for the first time in our studies).

**Appendix S6: Subgroup Comparison Analyses for the Psychopathy Subscales**

We explored possible differences in our psychopathy findings between the two subscales *callous-unemotional (CU) traits* and *impulsivity*. We expected that children in the predominantly proactive subgroup may display higher levels of CU traits but lower levels of impulsivity than children in the predominantly reactive subgroup. These analyses should be interpreted with caution, as we found below-acceptable internal consistencies of the subscales in Study 2 (*CU*: α = .48; *impulsivity*: α = .68) and in Study 3 for both parent-report (*CU*: α = .47; *impulsivity*: α = .56) and teacher-report (*CU*: α = .51; *impulsivity*: α = .53).

Table S8 describes the descriptive statistics for the *CU traits* and *impulsivity* scales, as well as for the total psychopathy scale reported in the manuscript. Findings were highly similar. With our main analyses, we found one significant subgroup difference for the total psychopathy scale in Study 3. With our supplementary analyses, we found that this difference is reflected only in the *CU traits* subscale, suggesting that the obtained effect for psychopathy in Study 3 may actually be driven by differences in children’s CU traits.

**Table S1**

*Fit Indices and Entropy for the Study 1-3 Confirmatory LPA Models Using Raw Scores and Different Ratios to Define the Subgroups*

| **Study 1** | **Ratio 1.25** | | | | **Ratio 1.50 (in paper)** | | | | **Ratio 1.75** | | | | **Ratio 2.00** | | | |
| --- | --- | --- | --- | --- | --- | --- | --- | --- | --- | --- | --- | --- | --- | --- | --- | --- |
|  | AIC | BIC | aBIC | Ent. | AIC | BIC | aBIC | Ent. | AIC | BIC | aBIC | Ent. | AIC | BIC | aBIC | Ent. |
| RE – pure | 982.15 | 1006.15 | 983.97 | .811 | 982.15 | 1006.15 | 983.97 | .811 | 982.15 | 1006.15 | 983.97 | .811 | 982.15 | 1006.15 | 983.97 | .811 |
| RE – pred. | 947.81 | 975.25 | 949.89 | .851 | 947.81 | 975.25 | 949.89 | .851 | 947.81 | 975.25 | 949.89 | .851 | 947.81 | 975.25 | 949.89 | .851 |
| Both – pure | 941.75 | 976.04 | 944.35 | .823 | 941.75 | 976.04 | 944.35 | .823 | 941.75 | 976.04 | 944.35 | .823 | 941.75 | 976.04 | 944.35 | .823 |
| Both – pred. | 904.67 | 945.82 | 907.79 | .874 | 904.69 | 945.84 | 907.81 | .875 | 905.27 | 946.42 | 908.39 | .876 | 906.96 | 948.11 | 910.08 | .876 |
| **Study 2** | **Ratio 1.25** | | | | **Ratio 1.50 (in paper)** | | | | **Ratio 1.75** | | | | **Ratio 2.00** | | | |
|  | AIC | BIC | aBIC | Ent. | AIC | BIC | aBIC | Ent. | AIC | BIC | aBIC | Ent. | AIC | BIC | aBIC | Ent. |
| RE – pure | 608.29 | 624.76 | 605.79 | .679 | 608.29 | 624.76 | 605.79 | .679 | 608.29 | 624.76 | 605.79 | .679 | 608.29 | 624.76 | 605.79 | .679 |
| RE – pred. | 586.45 | 605.66 | 583.53 | .751 | 585.93 | 605.14 | 583.02 | .744 | **585.93** | 605.14 | 583.02 | .744 | **586.00** | 605.21 | 583.09 | .745 |
| Both – pure | 601.56 | 623.52 | 598.24 | .844 | 601.56 | 623.52 | 598.24 | .844 | 601.56 | 623.52 | 598.24 | .844 | 601.56 | 623.52 | 598.24 | .844 |
| Both – pred. | 584.86 | 612.31 | 580.71 | **.850** | 585.92 | 613.37 | 581.76 | .793 | 586.42 | 613.87 | 582.26 | .802 | 587.21 | 614.66 | 583.05 | .813 |
| **Study 3** | **Ratio 1.25** | | | | **Ratio 1.50 (in paper)** | | | | **Ratio 1.75** | | | | **Ratio 2.00** | | | |
|  | AIC | BIC | aBIC | Ent. | AIC | BIC | aBIC | Ent. | AIC | BIC | aBIC | Ent. | AIC | BIC | aBIC | Ent. |
| RE – pure | 597.05 | 613.92 | 594.95 | - ^×^ | 597.05 | 613.92 | 594.95 | - ^×^ | 597.05 | 613.92 | 594.95 | - ^×^ | 597.05 | 613.92 | 594.95 | - ^×^ |
| RE – pred. | 583.01 | 602.69 | 580.56 | .751 | 583.01 | 602.69 | 580.56 | .751 | 583.01 | 602.69 | 580.56 | .751 | 583.01 | 602.69 | 580.56 | .751 |
| Both – pure | 600.56 | 623.06 | 597.76 | - ^×^ | 600.56 | 623.06 | 597.76 | - ^×^ | 600.56 | 623.06 | 597.76 | - ^×^ | 600.56 | 623.06 | 597.76 | - ^×^ |
| Both – pred. | 579.32 | 607.45 | 575.83 | .795 | 579.17 | 607.29 | 575.68 | .786 | 579.10 | 607.22 | 575.60 | .791 | 579.44 | 607.56 | 575.94 | .793 |

*Note*. Underlined values indicate best fit, and bold values indicate a ratio effect. Ent = Entropy. RE = Reactive-Only Models. Both = Both Subtypes Models. Pred = Predominant Models. ^×^No children were classified as purely reactive.**Table S2**

*Hypothesized Mean Differences (*H_A_*), Bootstrapped Significant Mean Differences (*M_diff_*), and Effect Sizes (η_p_^2^) of the Subgroup Comparison Analyses for Studies 1-3 Using Confirmatory LPA, Raw Scores, and Different Ratios to Define the Predominantly Reactive (R), Proactive (P) and Mixed (M) Subgroups*

| **Study 1** | | | | | | | | | | | |
| --- | --- | --- | --- | --- | --- | --- | --- | --- | --- | --- | --- |
|  |  |  | **Ratio 1.25** | | **Ratio 1.50** | | **Ratio 1.75** | | **Ratio 2.00** | | **Conclusion** |
|  |  | *H_A_* | *M_diff_* | η_p_^2^ | *M_diff_* | η_p_^2^ | *M_diff_* | η_p_^2^ | *M_diff_* | η_p_^2^ |  |
| Teacher-report | |  |  |  |  |  |  |  |  |  |  |
|  | ADHD symptoms | R,M>P | - | .03^*^ | M>R | .04^*^ | - | .05^*^ | M>R | .05^*^ | Same |
|  | Emotional symptoms | R,M>P | R>P | .06^*^ | R>P | .07^*^ | R>P | .06^*^ | R,M>P | .05^*^ | Same |
|  | Conduct problems | P,M>R | P,M>R | .09^*^ | M>R | .11^*^ | M>R | .10^*^ | M>R | .10^*^ | Same |
|  | Peer problems | R,M>P | - | <.01 | - | .02 | - | .01 | - | .01 | Same |
| Self-report | |  |  |  |  |  |  |  |  |  |  |
|  | Trait anxiety | R,M>P | - | .01 | - | .01 | - | .01 | - | .01 | Same |
|  | Psychopathy | P,M>R | - | <.01 | - | <.01 | - | <.01 | - | <.01 | Same |
|  | Dominance | P,M>R | - | <.01 | - | <.01 | - | <.01 | - | <.01 | Same |
|  | Empathy | P,M<R | - | .01 | - | .02 | - | .02 | - | .03^*^ | Same |
|  | Social acceptance | R,M<P | - | <.01 | - | .02 | - | .02 | - | .01 | Same |
| Social information processing | |  |  |  |  |  |  |  |  |  |  |
|  | Provoked anger | R,M>P | - | .02 | R>M | .04^*^ | - | .04^*^ | - | .03^*^ | Same |
|  | Hostile intent attribution | R,M>P | - | .03^*^ | R>M | .04^*^ | - | .04^*^ | - | .02 | Same |
|  | Aggression approval | P,M>R | - | <.01 | - | .01 | - | <.01 | - | <.01 | Same |
| Peer nomination | |  |  |  |  |  |  |  |  |  |  |
|  | Victimized | R,M>P | R>M,P | .04^*^ | R>M,P | .05^*^ | R>M,P | .05^*^ | R>M,P | .04^*^ | Same |
|  | Angry easily | R,M>P | - | .02 | - | .01 | - | <.01 | - | <.01 | Same |
|  | Social preference | R,M<P | - | .02 | - | .02 | - | .02 | - | .02 | Same |
|  | Popular | R,M<P | - | .02 | - | .01 | - | .02 | - | .03^*^ | Same |
|  | Coercive strategy use | P,M>R | M>R | .05^*^ | M>R | .04^*^ | M>R | .05^*^ | M>R | .07^*^ | Same |
|  | Bullies others | P,M>R | P>R | .07^*^ | P,M>R | .07^*^ | P,M>R | .08^*^ | P,M>R | .10^*^ | Same |

| **Study 2** | | | | | | | | | | | |
| --- | --- | --- | --- | --- | --- | --- | --- | --- | --- | --- | --- |
|  |  |  | **Ratio 1.25** | | **Ratio 1.50** | | **Ratio 1.75** | | **Ratio 2.00** | | **Conclusion** |
|  |  | *H_A_* | *M_diff_* | η_p_^2^ | *M_diff_* | η_p_^2^ | *M_diff_* | η_p_^2^ | *M_diff_* | η_p_^2^ |  |
|  | Psychopathy | P,M>R | - | .02 | - | .08 | - | .09^*^ | - | .09 | Same |
|  | Anger attribution bias | R,M>P | - | .02 | - | .03 | - | .02 | - | .02 | Same |
|  | Hostile intent attribution | R,M>P | - | <.01 | - | .03 | - | .03 | - | <.01 | Same |
| **Study 3** | | | | | | | | | | | |
|  |  |  | **Ratio 1.25** | | **Ratio 1.50** | | **Ratio 1.75** | | **Ratio 2.00** | | **Conclusion** |
|  |  | *H_A_* | *M_diff_* | η_p_^2^ | *M_diff_* | η_p_^2^ | *M_diff_* | η_p_^2^ | *M_diff_* | η_p_^2^ |  |
| Teacher-report | |  |  |  |  |  |  |  |  |  |  |
|  | ADHD symptoms | R,M>P | P,M>R | .09^*^ | P>R | .07^*^ | - | .08^*^ | - | .05^*^ | Same |
|  | Emotional symptoms | R,M>P | - | .02 | - | .02 | - | .02 | - | <.01 | Same |
|  | Conduct problems | P,M>R | M>R | .13^*^ | - | .07 | M>R | .10^*^ | M>R | .07^*^ | Different |
|  | Peer problems | R,M>P | M>R | .06^*^ | - | .03 | M>R | .07^*^ | - | .05^*^ | Same |
|  | Psychopathy | P,M>R | P,M>R | .11^*^ | P,M>R | .10^*^ | P,M>R | .14^*^ | M>R | .09^*^ | Same |
|  | Empathy | P,M<R | P,M<R | .22^*^ | P,M<R | .18^*^ | P,M<R | .19^*^ | P,M<R | .19^*^ | Same |
| Parent-report | |  |  |  |  |  |  |  |  |  |  |
|  | ADHD symptoms | R,M>P | - | <.01 | - | <.01 | - | .01 | - | .01 | Same |
|  | Emotional symptoms | R,M>P | - | <.01 | - | <.01 | - | <.01 | - | <.01 | Same |
|  | Conduct problems | P,M>R | - | .04 | - | .03 | - | .03 | - | .02 | Same |
|  | Peer problems | R,M>P | - | <.01 | - | .02 | - | .02 | - | .03 | Same |
|  | Psychopathy | P,M>R | - | <.01 | - | <.01 | - | .01 | - | .02 | Same |
|  | Empathy | P,M<R | - | .03 | - | .03 | - | .03 | - | .04 | Same |
| Empathic response | | P,M<R | - | .01 | - | .04 | - | .04 | - | .02 | Same |

*Note.* The column *H_A_* shows how the subgroups are predicted to deviate from each other. Underlined mean patterns at least partially support *H_A_*. Subgroups noted under *M_diff_* had non-overlapping bootstrap 95% CIs; subgroups excluded from *M_diff_* columns did not differ significantly from other subgroups. Effect sizes represent the overall subgroup effect and were based on parametric analyses (i.e., ANOVA). Hence, significance may differ between *M_diff_* and effect size columns, especially for larger ratios, which yielded smaller group sizes and therefore larger 95% CIs.

**Table S3**

*Fit Indices for the Hypothesized Models for Studies 1-3 Using Confirmatory LPA on Both Raw and Standardized Scores*

| **Study 1** | | | | | | | | |
| --- | --- | --- | --- | --- | --- | --- | --- | --- |
| **Raw Scores** | | | | | **Standardized Scores** | | | |
|  | AIC | BIC | aBIC | Entropy | AIC | BIC | aBIC | Entropy |
| Re. – pure | 982.15 | 1006.15 | 983.97 | .811 | 1254.41 | 1278.42 | 1256.23 | .811 |
| Re. – pred. | 947.81 | 975.25 | 949.89 | .851 | 1220.08 | **1247.51** | 1222.16 | **.851** |
| Both – pure | 941.75 | 976.04 | 944.35 | .823 | 1214.01 | 1248.31 | 1216.61 | .823 |
| Both – pred. | 904.69 | 945.84 | 907.81 | .875 | 1212.74 | 1253.89 | 1215.86 | .712 |
| **Study 2** | | | | | | | | |
| **Raw Scores** | | | | | **Standardized Scores** | | | |
|  | AIC | BIC | aBIC | Entropy | AIC | BIC | aBIC | Entropy |
| Re. – pure | 608.29 | 624.76 | 605.79 | .679 | 658.30 | 674.77 | 655.81 | .679 |
| Re. – pred. | 585.93 | 605.14 | 583.02 | .744 | 635.94 | 655.16 | 633.03 | .744 |
| Both – pure | 601.56 | 623.52 | 598.24 | .844 | 651.58 | 673.53 | 648.25 | .844 |
| Both – pred. | 585.92 | 613.37 | 581.76 | .793 | 633.06 | 660.51 | 628.90 | .840 |
| **Study 3** | | | | | | | | |
| **Raw Scores** | | | | | **Standardized Scores** | | | |
|  | AIC | BIC | aBIC | Entropy | AIC | BIC | aBIC | Entropy |
| Re. – pure | 597.05 | 613.92 | 594.95 | n.a.^×^ | 708.11 | 724.98 | 706.01 | n.a.^×^ |
| Re. – pred. | 583.01 | 602.69 | 580.56 | .751 | 694.07 | 713.76 | 691.62 | .751 |
| Both – pure | 600.56 | 623.06 | 597.76 | n.a.^×^ | 711.62 | 734.12 | 708.82 | n.a.^×^ |
| Both – pred. | 579.17 | 607.29 | 575.68 | .786 | 579.17 | **607.29** | 575.68 | .786 |

*Note*. Underlined values indicate best fit. Bold values indicate a standardization effect. RE = Reactive-Only Models. Both = Both Subtypes Models. Pred = Predominant Models.

^×^No children were classified as purely reactive.

**Table S4**

*Hypothesized Mean Differences (*H_A_*), Bootstrapped Significant Mean Differences* *(*M_diff_*), and Effect Sizes (η_p_^2^) of the Subgroup Comparison Analyses for Studies 1-3 Ran Using Confirmatory LPA, Raw and Standardized Scores, and Different Ratios to Define the Predominantly Reactive (R), Proactive (P) and Mixed (M) Subgroups*

| **Study 1** | | | | | | | | | | | | | | |
| --- | --- | --- | --- | --- | --- | --- | --- | --- | --- | --- | --- | --- | --- | --- |
|  |  |  | **Ratio 1.25**  **(*z*-scores)** | | **Ratio 1.50**  **(*z*-scores)** | | **Ratio 1.50**  **(raw scores)** | | **Ratio 1.75**  **(*z*-scores)** | | **Ratio 2.00**  **(*z*-scores)** | | **Conclusion** | |
|  |  | *H_A_* | *M_diff_* | η_p_^2^ | *M_diff_* | η_p_^2^ | *M_diff_* | η_p_^2^ | *M_diff_* | η_p_^2^ | *M_diff_* | η_p_^2^ | ***z* vs. raw** | **Ratios** |
| Teacher-report | |  |  |  |  |  |  |  |  |  |  |  |  |  |
|  | ADHD symptoms | R,M>P | - | .01 | - | .01 | M>R | .04^*^ | - | .01 | M>R | .04^*^ | Same | Same |
|  | Emotional symptoms | R,M>P | R>P | .07^*^ | R>P | .07^*^ | R>P | .07^*^ | R>P | .07^*^ | R,M>P | .09^*^ | Same | Same |
|  | Conduct problems | P,M>R | P>R | .05^*^ | P>R | .05^*^ | M>R,P | .11^*^ | P>R | .04 | P,M>R | .07^*^ | Same | Same |
|  | Peer problems | R,M>P | - | .01 | - | .01 | - | .02 | R>P | .04^*^ | R>P | .04^*^ | Same | Different |
| Self-report | |  |  |  |  |  |  |  |  |  |  |  |  |  |
|  | Trait anxiety | R,M>P | - | .02 | - | .01 | - | .01 | - | <.01 | - | .01 | Same | Same |
|  | Psychopathy | P,M>R | - | <.01 | - | <.01 | - | <.01 | - | .01 | - | <.01 | Same | Same |
|  | Dominance | P,M>R | - | <.01 | - | <.01 | - | <.01 | - | <.01 | - | <.01 | Same | Same |
|  | Empathy | P,M<R | - | <.01 | - | <.01 | - | .02 | - | .01 | - | .01 | Same | Same |
|  | Social acceptance | R,M<P | - | .02 | - | .02 | - | .02 | - | .03 | - | .04^*^ | Same | Same |
| Social information processing | |  |  |  |  |  |  |  |  |  |  |  |  |  |
|  | Provoked anger | R,M>P | - | .02 | - | .01 | R>M | .04^*^ | - | <.01 | - | .02 | Same | Same |
|  | Hostile intent attribution | R,M>P | - | .02 | - | <.01 | R>M | .04^*^ | - | <.01 | - | .01 | Same | Same |
|  | Aggression approval | P,M>R | - | <.01 | - | <.01 | - | .01 | - | <.01 | - | <.01 | Same | Same |
| Peer nomination | |  |  |  |  |  |  |  |  |  |  |  |  |  |
|  | Victimized | R,M>P | R>P | .06^*^ | R>P | .07^*^ | R>M,P | .05^*^ | R>P | .08^*^ | R>P | .07^*^ | Same | Same |
|  | Angry easily | R,M>P | - | <.01 | - | <.01 | - | .01 | - | <.01 | - | .02 | Same | Same |
|  | Social preference | R,M<P | - | <.01 | - | <.01 | - | .02 | - | .01 | M<R,P | .04^*^ | Same | Different |
|  | Popular | R,M<P | M<P,R | .03^*^ | - | .02 | - | .01 | - | .02 | - | .02 | Same | Different |
|  | Coercive strategy use | P,M>R | - | .03 | - | .03 | M>R | .04^*^ | - | .03^*^ | M>R | .05^*^ | Different | Different |
|  | Bullies others | P,M>R | P,M>R | .06^*^ | P>R | .06^*^ | P,M>R | .07^*^ | P>R | .06^*^ | P,M>R | .06^*^ | Same | Same |

| **Study 2** | | | | | | | | | | | | | | | | | | | | | | |
| --- | --- | --- | --- | --- | --- | --- | --- | --- | --- | --- | --- | --- | --- | --- | --- | --- | --- | --- | --- | --- | --- | --- |
|  |  |  | **Ratio 1.25**  **(*z*-scores)** | | **Ratio 1.50**  **(*z*-scores)** | | | **Ratio 1.50**  **(raw scores)** | | | **Ratio 1.75**  **(*z*-scores)** | | | | **Ratio 2.00**  **(*z*-scores)** | | | | **Conclusion** | | | |
|  |  | *H_A_* | *M_diff_* | η_p_^2^ | *M_diff_* | | η_p_^2^ | *M_diff_* | η_p_^2^ | | *M_diff_* | | η_p_^2^ | | *M_diff_* | | η_p_^2^ | | ***z* vs. raw** | | **Ratios** | |
| Psychopathy^×^ | | P,M>R | - | .05 | M>P | | .14^*^ | - | .08 | | M>R,P | | .13^*^ | | - | | .10^*^ | | Same | | Different | |
| Anger attribution bias^×^ | | R,M>P | - | .05 | - | | .03 | - | .03 | | - | | .06^*^ | | - | | .05 | | Same | | Same | |
| Hostile intent attribution^×^ | | R,M>P | - | <.01 | - | | .02 | - | .03 | | - | | .02 | | - | | .02 | | Same | | Same | |
| **Study 3** | | | | | | | | | | | | | | | | | | | | | | |
|  |  |  | **Ratio 1.25**  **(*z*-scores)** | | **Ratio 1.50**  **(*z*-scores)** | | | **Ratio 1.50**  **(raw scores)** | | | | **Ratio 1.75**  **(*z*-scores)** | | | | **Ratio 2.00**  **(*z*-scores)** | | | | **Conclusion** | | |
|  |  | *H_A_* | *M_diff_* | η_p_^2^ | *M_diff_* | η_p_^2^ | | *M_diff_* | | η_p_^2^ | | *M_diff_* | | η_p_^2^ | | *M_diff_* | | η_p_^2^ | | ***z* vs. raw** | | **Ratios** |
| Teacher-report | |  |  |  |  |  | |  | |  | |  | |  | |  | |  | |  | |  |
| ADHD symptoms | | R,M>P | - | .04 | P>R | .05^*^ | | P>R | | .07^*^ | | - | | .05 | | - | | .05 | | Same | | Same |
| Emotional symptoms | | R,M>P | - | .03 | - | .02 | | - | | .02 | | - | | .03 | | - | | .02 | | Same | | Same |
| Conduct problems | | P,M>R | - | .03 | - | .02 | | - | | .07 | | - | | .03 | | - | | .03 | | Same | | Same |
| Peer problems | | R,M>P | - | .01 | - | .01 | | - | | .03 | | - | | <.01 | | - | | <.01 | | Same | | Same |
| Psychopathy | | P,M>R | - | .05^*^ | - | .04 | | P,M>R | | .10^*^ | | - | | .04 | | - | | .05^*^ | | Different | | Same |
| Empathy | | P,M<R | P<R | .11^*^ | P<R | .10^*^ | | P,M<R | | .18^*^ | | P<R | | .09^*^ | | P<R | | .10^*^ | | Same | | Same |
| Parent-report | |  |  |  |  |  | |  | |  | |  | |  | |  | |  | |  | |  |
| ADHD symptoms | | R,M>P | - | <.01 | - | <.01 | | - | | <.01 | | - | | .01 | | - | | <.01 | | Same | | Same |
| Emotional symptoms | | R,M>P | - | <.01 | - | <.01 | | - | | <.01 | | - | | <.01 | | - | | .02 | | Same | | Same |
| Conduct problems | | P,M>R | - | <.01 | - | <.01 | | - | | .03 | | - | | <.01 | | - | | <.01 | | Same | | Same |
| Peer problems | | R,M>P | - | <.01 | - | <.01 | | - | | .02 | | - | | <.01 | | - | | <.01 | | Same | | Same |
| Psychopathy | | P,M>R | - | .02 | - | <.01 | | - | | <.01 | | - | | <.01 | | - | | .01 | | Same | | Same |
| Empathy | | P,M<R | M<R | .04 | - | .05 | | - | | .03 | | M<R | | .05^*^ | | M<R | | .07^*^ | | Same | | Different |
| Empathic response | | P,M<R | - | .02 | - | .02 | | - | | .04 | | - | | .02 | | - | | .02 | | Same | | Same |

*Note.* Findings from the paper (raw score columns) are included for comparison purposes. The column *H_A_* shows how the subgroups are predicted to deviate from each other. Underlined mean patterns at least partly support *H_A_*. Subgroups noted under *M_diff_* had non-overlapping bootstrap 95% CIs; subgroups excluded from *M_diff_* columns did not differ significantly from other subgroups. Effect sizes represent the overall subgroup effect and were based on parametric analyses (i.e., ANOVA). Hence, significance may differ between *M_diff_* and effect size columns, especially for larger ratios, which yielded smaller group sizes and therefore larger 95% CIs.

**Table S5**

*Fit Indices for the Study 1-3 Models Using Confirmatory LPA, Standardized Scores, and Different Ratios to Define the Subgroups*

| **Study 1** | **Ratio 1.25** | | | | **Ratio 1.50** | | | | **Ratio 1.75** | | | | **Ratio 2.00** | | | |
| --- | --- | --- | --- | --- | --- | --- | --- | --- | --- | --- | --- | --- | --- | --- | --- | --- |
|  | AIC | BIC | aBIC | Ent. | AIC | BIC | aBIC | Ent. | AIC | BIC | aBIC | Ent. | AIC | BIC | aBIC | Ent. |
| RE – pure | 1254.41 | 1278.42 | 1256.23 | .811 | 1254.41 | 1278.42 | 1256.23 | .811 | 1254.41 | 1278.42 | 1256.23 | .811 | 1254.41 | 1278.42 | 1256.23 | .811 |
| RE – pred. | 1220.08 | 1247.51 | 1222.16 | .851 | 1220.08 | 1247.51 | 1222.16 | .851 | 1220.08 | 1247.51 | 1222.16 | .851 | 1220.08 | 1247.51 | 1222.16 | .851 |
| Both – pure | 1214.01 | 1248.31 | 1216.61 | .823 | 1214.01 | 1248.31 | 1216.61 | .823 | 1214.01 | 1248.31 | 1216.61 | .823 | 1214.01 | 1248.31 | 1216.61 | .823 |
| Both – pred | 1212.73 | 1253.88 | 1215.85 | .711 | 1212.74 | 1253.89 | 1215.86 | .712 | 1212.74 | 1253.89 | 1215.86 | .712 | 1212.74 | 1253.89 | 1215.86 | .712 |
| **Study 2** | **Ratio 1.25** | | | | **Ratio 1.50** | | | | **Ratio 1.75** | | | | **Ratio 2.00** | | | |
|  | AIC | BIC | aBIC | Ent. | AIC | BIC | aBIC | Ent. | AIC | BIC | aBIC | Ent. | AIC | BIC | aBIC | Ent. |
| RE – pure | 658.30 | 674.77 | 655.81 | .679 | 658.30 | 674.77 | 655.81 | .679 | 658.30 | 674.77 | 655.81 | .679 | 658.30 | 674.77 | 655.81 | .679 |
| RE – pred. | 635.94 | 655.16 | 633.03 | .744 | 635.94 | 655.16 | 633.03 | .744 | 635.94 | 655.16 | 633.03 | .744 | 635.94 | 655.16 | 633.03 | .744 |
| Both – pure | 651.58 | 673.53 | 648.25 | .844 | 651.58 | 673.53 | 648.25 | .844 | 651.58 | 673.53 | 648.25 | .844 | 651.58 | 673.53 | 648.25 | .844 |
| Both – pred | 634.84 | 662.29 | 630.68 | .826 | 633.06 | 660.51 | 628.90 | .840 | 633.04 | 660.49 | 628.88 | **.844** | 635.85 | 663.30 | 631.69 | .626 |
| **Study 3** | **Ratio 1.25** | | | | **Ratio 1.50** | | | | **Ratio 1.75** | | | | **Ratio 2.00** | | | |
|  | AIC | BIC | aBIC | Ent. | AIC | BIC | aBIC | Ent. | AIC | BIC | aBIC | Ent. | AIC | BIC | aBIC | Ent. |
| RE – pure | 708.11 | 724.98 | 706.01 | n.a.^×^ | 708.11 | 724.98 | 706.01 | n.a.^×^ | 708.11 | 724.98 | 706.01 | n.a.^×^ | 708.11 | 724.98 | 706.01 | n.a.^×^ |
| RE – pred. | 694.07 | **713.76** | **691.62** | .751 | 694.07 | 713.76 | 691.62 | .751 | **694.07** | **713.76** | **691.62** | **.751** | **694.07** | **713.76** | **691.62** | **.751** |
| Both – pure | 711.62 | 734.12 | 708.82 | n.a.^×^ | 711.62 | 734.12 | 708.82 | n.a.^×^ | 711.62 | 734.12 | 708.82 | n.a.^×^ | 711.62 | 734.12 | 708.82 | n.a.^×^ |
| Both – pred | 700.07 | 728.19 | 696.57 | .843 | 579.17 | 607.29 | 575.68 | .786 | 696.73 | 724.85 | 693.23 | .694 | 696.88 | 725.00 | 693.38 | .695 |

*Note*. Ent = Entropy. RE = Reactive-Only Models. Both = Both Subtypes Models. Pred = Predominant Models. Underlined values indicate best fit. Bold values indicate a ratio effect. ^×^No children were classified as purely reactive.

**Table S6**

*Fit Indices for the Study 1-3 Models Using Exploratory LPA*

| **Study 1** | | | | |
| --- | --- | --- | --- | --- |
|  | AIC | BIC | aBIC | Entropy |
| 1-class model | 1027.80 | 1041.52 | 1028.84 | – |
| 2-class model | 947.81 | 975.25 | 949.89 | .851 |
| 3-class model | 904.64 | 945.79 | 907.76 | .875 |
| 4-class model | 863.56 | 918.43 | 867.72 | .934 |
| **Study 2** | | | | |
|  | AIC | BIC | aBIC | Entropy |
| 1-class model | 608.69 | 619.67 | 607.03 | – |
| 2-class model | 585.93 | 605.14 | 583.02 | .744 |
| 3-class model | 582.63 | 610.08 | 578.48 | .842 |
| 4-class model | 577.02 | 612.71 | 571.62 | .750 |
| **Study 3** | | | | |
|  | AIC | BIC | aBIC | Entropy |
| 1-class model | 593.05 | 604.30 | 591.65 | – |
| 2-class model | 583.01 | 602.69 | 580.56 | .751 |
| 3-class model | 579.06 | 607.18 | 575.56 | .789 |
| 4-class model | 570.43 | 606.99 | 565.88 | .860 |

*Note*. Underlined values indicate best fit.

**Table S7**

*Hypothesized Mean Differences (*H_A_*), Bootstrapped Significant Mean Differences (*M_diff_*), and Effect Sizes (η_p_^2^) of the Subgroup Comparison Analyses for Studies 1-3 Using Subgroups of Mildly Reactive (R~), Strongly Reactive (R+), Mildly Proactive (P~), Strongly Proactive (P+) and Mixed (M) children Formed Through Confirmatory versus Exploratory LPA*

| **Study 1** | | | | | | | |
| --- | --- | --- | --- | --- | --- | --- | --- |
|  |  |  | **Confirmatory** | | **Exploratory** | | **Conclusion** |
|  |  | *H_A_* | *M_diff_* | η_p_^2^ | *M_diff_* | η_p_^2^ |  |
| Teacher-report | |  |  |  |  |  |  |
|  | ADHD symptoms | R,M>P | M>R | .04^*^ | P+>R,M,P~ | .10^*^ | Same |
|  | Emotional symptoms | R,M>P | R>P | .07^*^ | - | .02 | Different |
|  | Conduct problems | P,M>R | M>R,P | .11^*^ | P+>P~>R,M | .27^*^ | Same |
|  | Peer problems | R,M>P | - | .02 | - | .02 | Same |
| Self-report | |  |  |  |  |  |  |
|  | Trait anxiety | R,M>P | - | .01 | - | .01 | Same |
|  | Psychopathy | P,M>R | - | <.01 | - | <.01 | Same |
|  | Dominance | P,M>R | - | <.01 | - | .02 | Same |
|  | Empathy | P,M<R | - | .02 | - | <.01 | Same |
|  | Social acceptance | R,M<P | - | .02 | - | <.01 | Same |
| Social information processing | |  |  |  |  |  |  |
|  | Provoked anger | R,M>P | R>M | .04^*^ | - | .01 | Same |
|  | Hostile intent attribution | R,M>P | R>M | .04^*^ | - | .03 | Same |
|  | Aggression approval | P,M>R | - | .01 | - | .02 | Same |
| Peer nomination | |  |  |  |  |  |  |
|  | Victimized | R,M>P | R>M,P | .05^*^ | - | <.01 | Different |
|  | Angry easily | R,M>P | - | .01 | P+>R | .06^*^ | Same |
|  | Social preference | R,M<P | - | .02 | P+<R | .09^*^ | Same |
|  | Popular | R,M<P | - | .01 | - | .02 | Same |
|  | Coercive strategy use | P,M>R | M>R | .04^*^ | P~,P+>R,M | .12^*^ | Same |
|  | Bullies others | P,M>R | P,M>R | .07^*^ | P~,P+>R,M | .14^*^ | Same |
| **Study 2** | | | | | | | |
|  |  |  | **Confirmatory** | | **Exploratory** | | **Conclusion** |
|  |  | *H_A_* | *M_diff_* | η_p_^2^ | *M_diff_* | η_p_^2^ |  |
|  | Psychopathy^×^ | P,M>R | - | .08 | R>M | .10^*^ | Same |
|  | Anger attribution bias^×^ | R,M>P | - | .03 | - | .06^*^ | Same |
|  | Hostile intent attribution^×^ | R,M>P | - | .03 | - | .01 | Same |
| **Study 3** | | | | | | | |
|  |  |  | **Confirmatory** | | **Exploratory** | | **Conclusion** |
|  |  | *H_A_* | *M_diff_* | η_p_^2^ | *M_diff_* | η_p_^2^ |  |
| Teacher-report | |  |  |  |  |  |  |
|  | ADHD symptoms | R,M>P | P>R | .07^*^ | R~,P>R+ | .14^*^ | Same |
|  | Emotional symptoms | R,M>P | - | .02 | - | .01 | Same |
|  | Conduct problems | P,M>R | - | .07 | R~,M>R+ | .18^*^ | Different |
|  | Peer problems | R,M>P | - | .03 | - | .05 | Same |
|  | Psychopathy | P,M>R | P,M>R | .10^*^ | R~,P,M>R+ | .24^*^ | Same |
|  | Empathy | P,M<R | P,M<R | .18^*^ | P<M,R~<R+ | .18^*^ | Same |
| Parent-report | |  |  |  |  |  |  |
|  | ADHD symptoms | R,M>P | - | <.01 | - | .05 | Same |
|  | Emotional symptoms | R,M>P | - | <.01 | - | <.01 | Same |
|  | Conduct problems | P,M>R | - | .03 | - | .04 | Same |
|  | Peer problems | R,M>P | - | .02 | - | .02 | Same |
|  | Psychopathy | P,M>R | - | <.01 | - | .03 | Same |
|  | Empathy | P,M<R | - | .03 | M< R~,R+ | .09^*^ | Different |
| Empathic response | | P,M<R | - | .04 | - | .02 | Same |

*Note.* The column *H_A_* shows how the subgroups are predicted to deviate from each other. Underlined mean patterns at least partly support *H_A_*. Subgroups noted under *M_diff_* had non-overlapping bootstrap 95% CIs; subgroups excluded from *M_diff_* columns did not differ significantly from other subgroups. Effect sizes represent the overall subgroup effect and were based on parametric analyses (i.e., ANOVA). Hence, significance may differ between *M_diff_* and effect size columns, especially for larger ratios, which yielded smaller group sizes and therefore larger 95% CIs.

**Table S8**

*Hypothesized Mean Differences (*H_A_*), Range, Means (*M*), and Standard Deviations (*SD*) of the Total Psychopathy Scale, the CU Traits Subscale, and the Impulsivity Subscale for the Predominantly Reactive (R), Proactive (P) and Mixed (M) Subgroups for Studies 1-3*

| Study/ reporter |  |  |  | Reactive | | Mixed | | Proactive | |  |
| --- | --- | --- | --- | --- | --- | --- | --- | --- | --- | --- |
|  | Scale | *H_A_* | Range | *M* | *SD* | *M* | *SD* | *M* | *SD* | η_p_^2^ |
| 1/self^×^ | Psychopathy | P,M>R | 0.00-2.35 | 0.61 | 0.39 | 0.57 | 0.46 | 0.63 | 0.50 | <.01 |
|  | CU traits | P,M>R | 0.00-3.00 | 0.66 | 0.57 | 0.58 | 0.60 | 0.65 | 0.59 | <.01 |
| 2/teacher | Psychopathy | P,M>R | 0.25-1.65 | 0.80 | 0.31 | 1.00 | 0.37 | 0.99 | 0.47 | .08 |
|  | CU traits | P,M>R | 0.00-1.67 | 0.76 | 0.36 | 0.83 | 0.29 | 1.00 | 0.52 | .04 |
|  | Impulsivity | R,M>P | 0.20-2.00 | 1.12 | 0.47 | 1.25 | 0.50 | 0.93 | 0.59 | .03 |
| 3/teacher | Psychopathy | P,M>R | 0.05-1.55 | 0.58^a^ | 0.30 | 0.75^b^ | 0.29 | 0.83^b^ | 0.32 | .10^*^ |
|  | CU traits | P,M>R | 0.00-1.67 | 0.74^a^ | 0.38 | 0.85^ab^ | 0.27 | 1.05^b^ | 0.33 | .10^*^ |
|  | Impulsivity | R,M>P | 0.00-1.80 | 0.78 | 0.39 | 0.95 | 0.37 | 0.88 | 0.40 | .03 |
| 3/parent | Psychopathy | P,M>R | 0.20-1.65 | 0.81 | 0.32 | 0.82 | 0.32 | 0.76 | 0.26 | <.01 |
|  | CU traits | P,M>R | 0.00-1.67 | 0.71 | 0.32 | 0.80 | 0.34 | 0.82 | 0.38 | .02 |
|  | Impulsivity | R,M>P | 0.00-2.00 | 1.14 | 0.44 | 1.18 | 0.36 | 1.03 | 0.42 | .01 |

*Note.* Column *H_A_* shows how the subgroups are predicted to deviate from each other. Underlined predictions were supported. Subgroups with different superscripts differed significantly. ^×^Study 1 data collection did not include the impulsivity subscale.
